# Robust, persistent adaptive immune responses to SARS-CoV-2 in the oropharyngeal lymphoid tissue of children

**DOI:** 10.21203/rs.3.rs-1276578/v1

**Published:** 2022-03-23

**Authors:** Qin Xu, Pedro Milanez-Almeida, Andrew J. Martins, Andrea J. Radtke, Kenneth B. Hoehn, Jinguo Chen, Can Liu, Juanjie Tang, Gabrielle Grubbs, Sydney Stein, Sabrina Ramelli, Juraj Kabat, Hengameh Behzadpour, Maria Karkanitsa, Jacquelyn Spathies, Heather Kalish, Lela Kardava, Martha Kirby, Foo Cheung, Silvia Preite, Patrick C. Duncker, Nahir Romero, Diego Preciado, Lyuba Gitman, Galina Koroleva, Grace Smith, Arthur Shaffer, Ian T. McBain, Stefania Pittaluga, Ronald N. Germain, Richard Apps, Kaitlyn Sadtler, Susan Moir, Daniel S. Chertow, Steven H. Kleinstein, Surender Khurana, John S. Tsang, Pamela Mudd, Pamela L. Schwartzberg, Kalpana Manthiram

**Affiliations:** 1 Cell Signaling and Immunity Section, Laboratory of Immune System Biology (LISB), National Institute of Allergy and Infectious Diseases (NIAID), National Institutes of Health (NIH), Bethesda, MD; 2 Center for Human Immunology, NIAID, NIH, Bethesda, MD; 3 Multiscale Systems Biology Section, LISB, NIAID, NIH, Bethesda, MD; 4 Center for Advanced Tissue Imaging, LISB, NIAID, NIH Bethesda, MD; 5 Department of Pathology, Yale School of Medicine, New Haven, CT; 6 Division of Viral Products, Center for Biologics Evaluation and Research (CBER), Food and Drug Administration (FDA), Silver Spring, MD; 7 Emerging Pathogens Section, Critical Care Medicine Department, Clinical Center (CC), NIH, Bethesda, MD; 8 Laboratory of Immunoregulation, NIAID, NIH, Bethesda, MD; 9 Division of Pediatric Otolaryngology, Children’s National Hospital, Washington, DC; 10 Laboratory of Immuno-Engineering, National Institute of Biomedical Imaging and Bioengineering (NIBIB), NIH, Bethesda, MD; 11 Trans-NIH Shared Resource on Biomedical Engineering and Physical Science, NIBIB, NIH, Bethesda, MD; 12 B-cell Immunology Section, Laboratory of Immunoregulation, NIAID, NIH, Bethesda, MD; 13 National Human Genome Research Institute (NHGRI), NIH, Bethesda, MD; 14 Cytek Biosciences, Fremont, CA; 15 Division of Otolaryngology, Department of Surgery, George Washington University School of Medicine and Health Sciences, Washington, DC; 16 Laboratory of Pathology, Center for Cancer Research, National Cancer Institute (NCI), NIH, Bethesda, MD; 17 Lymphoid Malignancies Branch, Center for Cancer Research, NCI, NIH, Bethesda, MD; 18 Lymphocyte Biology Section, LISB, NIAID, NIH, Bethesda, MD; 19 Program in Computational Biology and Bioinformatics, Yale University, New Haven, CT; 20 Department of Immunobiology, Yale School of Medicine, New Haven, CT

## Abstract

SARS-CoV-2 infection triggers adaptive immune responses from both T and B cells. However, most studies focus on peripheral blood, which may not fully reflect immune responses in lymphoid tissues at the site of infection. To evaluate both local and systemic adaptive immune responses to SARS-CoV-2, we collected peripheral blood, tonsils, and adenoids from 110 children undergoing tonsillectomy/adenoidectomy during the COVID-19 pandemic and found 24 with evidence of prior SARS-CoV-2 infection, including detectable neutralizing antibodies against multiple viral variants. We identified SARS-CoV-2-specific germinal center (GC) and memory B cells; single cell BCR sequencing showed that these virus-specific B cells were class-switched and somatically hypermutated, with overlapping clones in the adenoids and tonsils. Oropharyngeal tissues from COVID-19-convalescent children showed persistent expansion of GC and anti-viral lymphocyte populations associated with an IFN-γ-type response, with particularly prominent changes in the adenoids, as well as evidence of persistent viral RNA in both tonsil and adenoid tissues of many participants. Our results show robust, tissue-specific adaptive immune responses to SARS-CoV-2 in the upper respiratory tract of children weeks to months after acute infection, providing evidence of persistent localized immunity to this respiratory virus.

## Introduction

SARS-CoV-2 induces humoral and cellular immune responses in children, primarily noted by assessing antibody and T cell responses in the peripheral blood^1,2^. However, little is known about immune responses to the virus in the lymphoid tissue of the upper respiratory tract where initial infection and viral replication take place^3,4^. The palatine tonsils and adenoids are secondary lymphoid structures at the mucosal surface of the naso- and oropharynx, where tissue-specific T and B cell responses to antigens in the upper respiratory tract can be generated^5,6^. Here, collaborative interactions between T follicular helper cells (Tfh) and B cells enable immunoglobulin gene class switching and formation of germinal centers (GCs), where B cells undergo somatic hypermutation of immunoglobulin genes that supports affinity maturation, resulting in the production of high-affinity antibodies and memory B cells. In adults with fatal COVID-19, loss of GCs in draining thoracic lymph nodes and consequentially, poor serum antibody durability have been reported; however, recently, others have found evidence of durable B cell responses derived from GCs including long-lived plasma cells in the bone marrow of convalescent adults as well as antigen-specific GC B cells and Tfh cells in the lymph nodes and lung tissues of organ donors^7–13^. As tonsillectomy and adenoidectomy are among the most common ambulatory surgeries in children, the tonsils and adenoids offer an accessible secondary lymphoid tissue enabling the study of GC and T cell responses to SARS-CoV-2 in children^14^. Using in-depth immune profiling, we characterized adaptive immune responses to SARS-CoV-2 in the tonsils and adenoids of convalescent children and described long-term alterations in tissue-specific B and T lymphocyte populations involved in GC and anti-viral memory responses following COVID-19.

## Robust GC responses in pharyngeal lymphoid tissue

**Figure 1.**
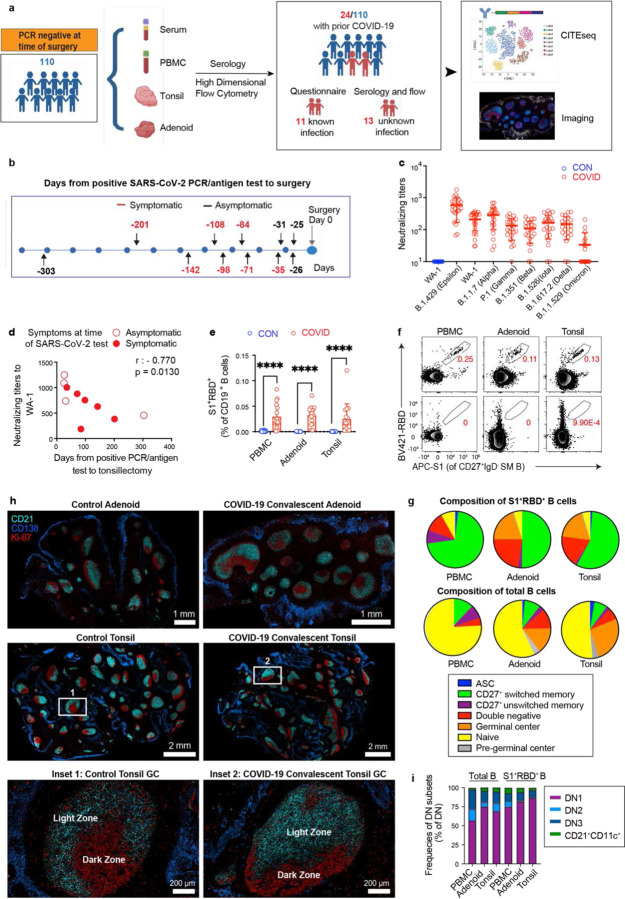
SARS-CoV-2 elicits robust humoral immune responses in children a. Participant enrollment and study design. b. Time from positive SARS-CoV-2 PCR/antigen test from nasopharyngeal swab to tonsillectomy and/or adenoidectomy surgery. c. Neutralization titers (PsVNA50) against the early isolate WA-1 and seven other SARS-CoV-2 variants of interest in COVID-19 convalescent subjects (COVID) vs. controls (CON) (COVID n=23, CON n=14, samples listed in Supplementary Table 4). d. Correlation between neutralizing antibody titers to WA-1 and days from positive SARS-CoV-2 test to surgery (n = 10). e. Frequency of S1^+^RBD^+^ cells among total CD19^+^ B cells from PBMC, adenoid, and tonsil from COVID vs. CON (PBMC COVID n = 18, CON n = 33; adenoid COVID n = 16, CON n = 27; and tonsil COVID n = 16, CON n = 30). f. Representative flow cytometry plots demonstrating the percentage of SARS-CoV-2-specific (S1^+^RBD^+^) cells among CD27^+^IgD^−^ switched memory B cells in PBMC, adenoid, and tonsil following COVID-19. Gating strategy shown in Supplementary Fig 1–2. g. Composition of S1^+^RBD^+^ B cells and total B cells from PBMC, adenoid, and tonsil from COVID-19 convalescent subjects. Mean frequency of each B cell subset is presented in the pie chart. B cell subsets are defined in Supplementary Fig 1–2. ASC = antibody secreting cells, equivalent to plasma cells and plasmablasts. h. Representative images of adenoid and tonsil from a COVID-19-convalescent donor showing multiple, intact germinal centers (GCs) comparable to that from controls. Inset shows close-up of GC with discrete light and dark zones. CD21 (follicular dendritic cells, light zone) in cyan, Ki-67 (dividing cells, dark zone) in red, CD138 (plasma cells and epithelial cell marker) in blue. i. Composition of S1^+^RBD^+^ double negative (DN) B cells and total DN B cells from PBMC, adenoid, and tonsil (COVID PBMC n = 18, adenoid n = 16, tonsil n = 16). Mean frequency of each DN subset is presented in the bar chart. Each symbol represents data from one donor. Means ± S.D. are displayed in the scatter and bar plots. Significance calculated with Mann-Whitney U test. Correlations assessed with Spearman’s rank correlation. **** p<0.0001.

We collected blood, tonsils, and adenoids from 110 children who underwent tonsillectomy and/or adenoidectomy primarily from September 2020 to January 2021 (Fig. 1a, participant characteristics in Supplementary Tables 1–3). All participants were required to have a negative PCR for SARS-CoV-2 from a nasopharyngeal swab within 72 hours prior to surgery. Eleven participants had histories of confirmed SARS-CoV-2 infection by PCR or antigen detection from previous nasopharyngeal swabs, ranging from 25 to 303 days prior to surgery (average 102 days); 64% (7/11) of these participants reported symptoms at the time of positive testing (Fig. 1b, Supplementary Table 3). Thirteen additional participants with previous SARS-CoV-2 infection were identified after sample collection through serological testing and/or identification of B cells that bind probes for both the S1 domain of the spike protein (S1) and spike receptor binding domain (RBD) from SARS-CoV-2 (S1^+^RBD^+^), yielding a total of 24 participants with evidence of prior COVID-19 in our cohort (Fig. 1a, Supplementary Table 4). Neutralizing antibodies against the WA-1, B.1.1.7 (alpha), and B.1.429 (epsilon) strains were detected in the serum of all seropositive subjects but not in controls (Fig. 1c, Supplementary Table 4). Most seropositive subjects also had neutralizing antibodies to other strains including B.1.617.2 (delta), although fewer (9 out of 23) had neutralizing antibodies to B.1.1.529 (omicron) (Fig. 1c, Supplementary Table 4). Neutralizing titers were highest against the WA-1 strain and inversely correlated with time since positive PCR/antigen test in those participants with prior testing (Fig. 1d).

In nearly all seropositive participants, we detected S1^+^RBD^+^ B cells in PBMCs and both pharyngeal tissues (Fig. 1e), with the exception of two donors (CNMC 91 and 104) who had almost no S1^+^RBD^+^ binding B cells in the peripheral blood (Extended Data Fig. 1a). These two donors also had the lowest serum neutralizing antibody titers to WA-1 among our cohort. Surprisingly, one participant (CNMC 32) had high serum neutralization titers but very low percentages of S1^+^RBD^+^ B cells, particularly in the oropharyngeal tissues, highlighting heterogeneity in responses to SARS-CoV-2.

Evaluation of B cell populations by high-dimensional flow cytometry revealed that the majority of S1^+^RBD^+^ B cells were CD27^+^ immunoglobulin (Ig) class-switched memory B cells (IgD^−^CD38^−^CD27^+^) (Fig. 1f–g, Extended Data Fig. 1b, Supplementary Fig. 1–2), indicating a robust memory B cell response was generated and maintained in the upper respiratory tract as long as 10 months into the convalescent period (Extended Data Fig. 1c). These S1^+^RBD^+^ memory B cells were primarily IgG^+^, with lower percentages of IgA^+^ cells compared to total CD27^+^ memory B cells in the tissue, perhaps reflecting the inflammatory milieu during infection (Extended Data Fig. 1d). Of note, the percentage of S1^+^RBD^+^ cells we found among CD27^+^ switched memory B cells in the oropharyngeal tissue was comparable to that recently reported in lung and lung-draining lymph nodes from convalescent autopsy donors (Fig. 1f, Extended Data Fig. 1e)^13^.

The predominance of Ig class-switched CD27^+^ memory B cells among S1^+^RBD^+^ B cells suggested that they originated from GC reactions, although the timing of class switching remains controversial^15^. Because the tonsils and adenoids are secondary lymphoid tissues and sites of robust GC formation, we could directly examine the involvement of GCs. Flow cytometric analysis revealed a substantial portion of GC B cells among the S1^+^RBD^+^ B cells in both tissues (Fig. 1g). Paired analyses of tonsils and adenoids from the same donor revealed that the adenoids had higher frequencies of S1^+^RBD^+^ cells among both total and GC B cells compared to tonsils, perhaps reflecting higher viral exposure due to their location in the nasopharynx (Extended Data Fig. 1f–g). Frequencies of S1^+^RBD^+^ B cells in the adenoids, but not tonsils or PBMCs, correlated significantly with serum neutralization titers for B.1.351 (beta), B.1.526 (iota), B.1.617.2 (delta), and B.1.1.529 (omicron) variants, further highlighting the importance of the adenoids in generating immune responses to SARS-CoV-2 (Extended Data Fig. 1h). Furthermore, in contrast to previous reports of absent GC structures in secondary lymphoid organs in postmortem analyses of adults who died from severe COVID-19^7^, we observed intact GC structures in both adenoid and tonsil tissue of children following COVID-19 using multiplex immunofluorescence microscopy, with discrete dark and light zones; COVID-19-convalescent tissues did not exhibit smaller or fewer GCs relative to tissues from uninfected controls (Fig. 1h; Extended Data Fig. 1i–j).

Early responses to SARS-CoV-2 in symptomatic patients have been shown to be dominated by extrafollicular responses, characterized by expansion of IgD^−^CD27^−^ double negative (DN, IgD^−^CD27^−^CD38^−^CD19^+^) B cells^16,17^. We also saw an expansion of DN B cells among S1^+^RBD^+^ B cells in both adenoids and tonsils (Fig. 1g). However, most of these S1^+^RBD^+^ DN B cells exhibited characteristics of DN1 (CD21^+^CD11c^−^) cells, which are derived from GCs (Fig. 1i). Only a small portion were DN2 (CD21^−^CD11c^+^) cells, which originate from extrafollicular B cell activation and were reported to expand in acute severe COVID-19^16^. Our findings, therefore, suggest that robust humoral responses to SARS-CoV-2 associated with intact GC reactions and B cell memory are present in the upper respiratory tract mucosal lymphoid tissue.

## Multimodal single-cell analysis of SARS-CoV-2-specific B cells

**Extended Data Figure 2.**
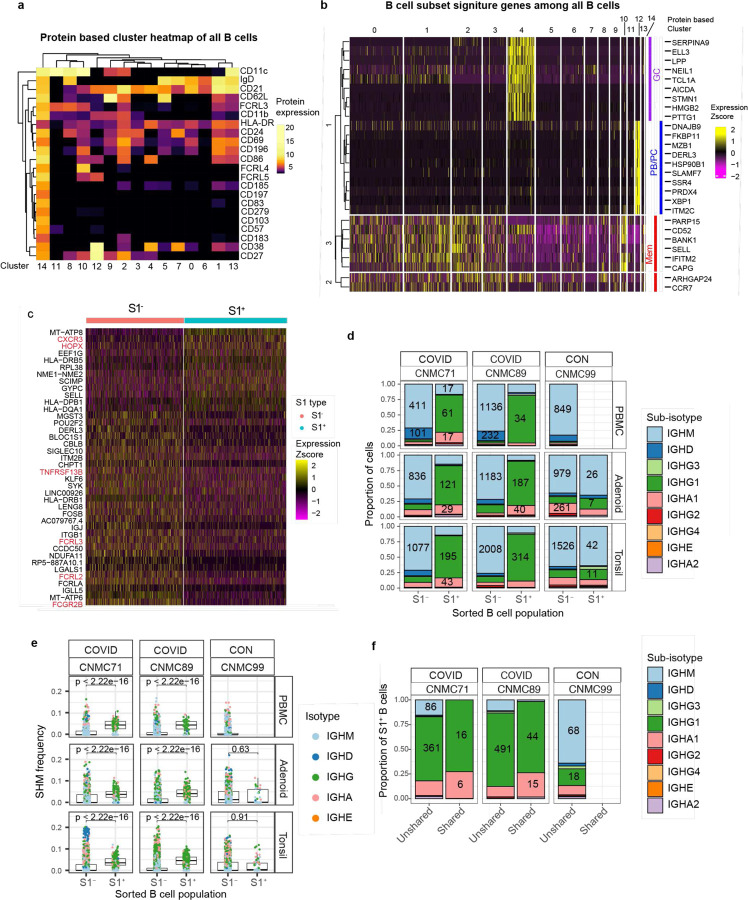
CITE-seq analysis of SARS-CoV-2 antigen-specific B cells a. Heatmap of unsupervised clustering by CITE-seq antibody expression of S1^+^ and S1^−^ B cells from tonsil, adenoid, and PBMCs from three donors (2 COVID-19 convalescent and 1 control) yielding 15 clusters. Most S1^+^ B cells are in cluster 2 which is IgD^−^ and CD27^+^, indicative of a memory B cell phenotype. b. Heatmap showing expression of signature gene sets for germinal center (GC) B cells, memory B cells, and plasma cells/plasmablasts (PC/PB) among all B cells (S1^+^ and S1^−^) from tonsil, adenoid, and PBMC organized by cluster. c. Heatmap showing differentially expressed (DE) genes in S1^+^ vs. S1^−^ B cells from tonsil and adenoid from cluster 2 (which are CD27^+^ memory B cells, shown in Fig. 2a and Extended Data Fig. 2a). DE gene list is in Supplementary Table 5. d. Sub-isotype frequencies among S1^+^ and S1^−^ B cells from adenoid, tonsil and PBMC of two COVID-19 convalescent donors (CNMC 71 and 89) and one control (CNMC 99). Labels show the raw number of cells with a given sub-isotype and are only shown for sub-isotypes that make up at least 10% of a given category. e. Somatic hypermutation (SHM) frequency among S1^+^ and S1^−^ B cells of all isotypes from PBMC, adenoid, and tonsil of each donor. Mutation frequency calculated in V gene. Significance calculated with the Mann Whitney U test. f. Sub-isotype frequencies among S1^+^ B cells from clones shared between tonsil and adenoid vs. unshared clones. Labels show the raw number of cells with a given sub-isotype and are only included for sub-isotypes that make up at least 10% of a given category.

**Extended Data Figure 3.**
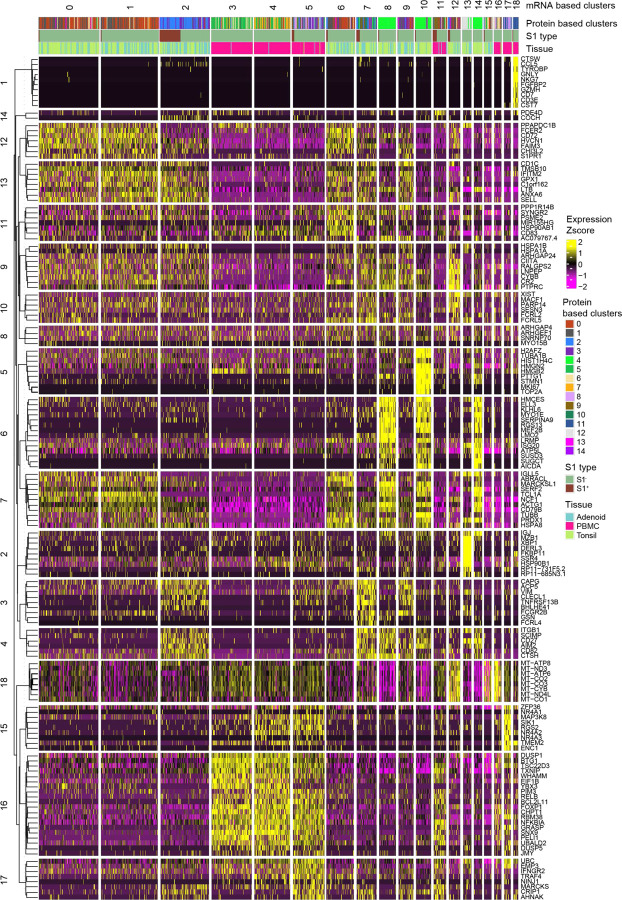
Gene-based clustering of CITE-seq of S1^+^ and S1^−^ B cells Unsupervised clustering based on gene expression of sorted S1^+^ and S1^−^ B cells from tonsil, adenoid, and PBMCs from three donors (2 COVID-19 convalescent and 1 control) yielding 19 clusters. Top defining genes for each cluster are noted. Top bar shows the corresponding cluster based on CITE-seq surface protein expression (shown in Extended Data Fig. 2a–b); middle bar indicates which cells are S1^+^, and lower bar indicates tissue of origin.

**Figure 2.**
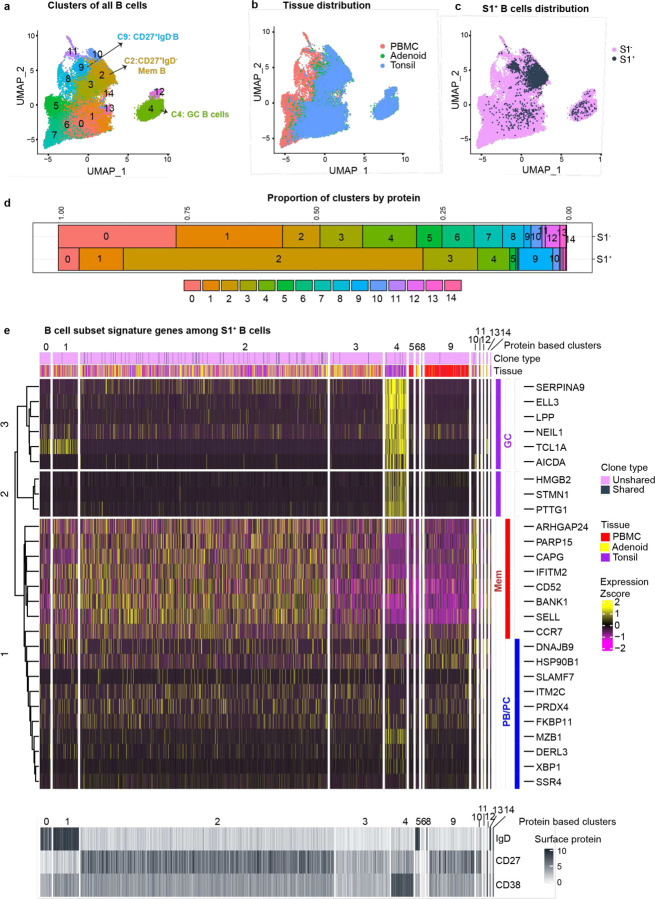
CITE-seq analysis of SARS-CoV-2 antigen-specific B cells a. Uniform manifold approximation and projection (UMAP) showing 15 clusters of sorted S1^+^ and S1^−^ B cells from tonsil, adenoid, and PBMCs of three donors (2 COVID-19-convalescent and 1 control) clustered according to CITE-seq surface antibody expression. b and c. Tissue distribution of cells is shown in b. S1^+^ B cells are highlighted c. d. Proportion of each cluster among S1^−^ and S1^+^ B cells. e. Heat map showing expression of signature gene sets for germinal center B cells (GC), memory B cells(Mem), and plasma cells/plasmablasts (PB/PC)^18^ among S1^+^ B cells organized by cluster. IgD, CD38, and CD27 CITE-seq antibody expression are shown in lower heat map in grey. Tissue origin is shown in purple (tonsil), yellow (adenoid), and red (PBMC), while clones shared between tonsil and adenoid are marked in black in the top bar. Sorting strategy shown in Supplementary Fig. 3.

To investigate B cell responses in greater detail, we sorted S1-binding (S1^+^) and non-binding (S1^−^) B cells from tonsils, adenoids, and PBMCs from two subjects with a history of COVID-19, as well as one uninfected control (Supplementary Fig. 3a–b). Over 1860 S1^+^ B cells and 25000 S1^−^ B cells were captured and characterized by CITE-seq (Cellular Indexing of Transcriptomes and Epitopes by Sequencing), which simultaneously measured the expression of 22 B cell surface markers and sequenced the transcriptome and V(D)J/BCR in single cells. We performed unsupervised clustering using cell surface protein expression profiles (Fig. 2a–d, Extended Data Fig. 2a) and assessed the expression of memory B cell, GC B cell, and plasma cell/plasmablast transcriptional signatures in each cluster^18^ (Fig. 2e, Extended Data Fig. 2b). Surface antibody staining patterns were concordant with the cell types suggested by the gene expression signatures in each cluster (S1^+^ B cells in Fig. 2e, total B cells in Extended Data Fig. 2a–b). Consistent with our flow cytometric analysis, the majority of S1^+^ B cells in the tonsils and adenoids were in cluster 2, which represented CD27^+^ memory B cells (Fig. 2c–e). Adenoids and tonsils had a smaller but clear portion of S1^+^ cells that were in cluster 4, which had a GC B cell gene expression signature and surface protein profile (Fig. 2b–e, Extended Data Fig. 2a–b). In contrast, S1^+^ cells in the blood were primarily in cluster 9 (Fig. 2a–c, e), which was also a CD27^+^IgD^−^ population (Fig. 2e, lower heatmap) but had different surface marker and gene expression profiles compared to CD27^+^IgD^−^ memory B cells in the lymphoid tissues (Fig. 2e, upper heatmap; Extended Data Fig. 2a). S1^+^ cells from both the peripheral blood and tissues also clustered separately when cells were clustered by transcript expression alone (Extended Data Fig. 3). Furthermore, S1^+^ memory B cells in cluster 2 had higher expression of *CXCR3* and *HOPX*, genes known to be induced by T-bet in T cells^19^, than their S1^−^ counterparts, suggesting that they may have developed in a more IFN-γ rich environment (Supplementary Table 5, Extended Data Fig. 2c). These S1^+^ memory B cells also had decreased expression of several regulatory receptors that inhibit BCR signaling including *FCGR2B, FCRL2, FCRL3,* and *TNFRSF13B* (encoding TACI)^20,21^ (Supplementary Table 5, Extended Data Fig. 2c).

**Figure 3.**
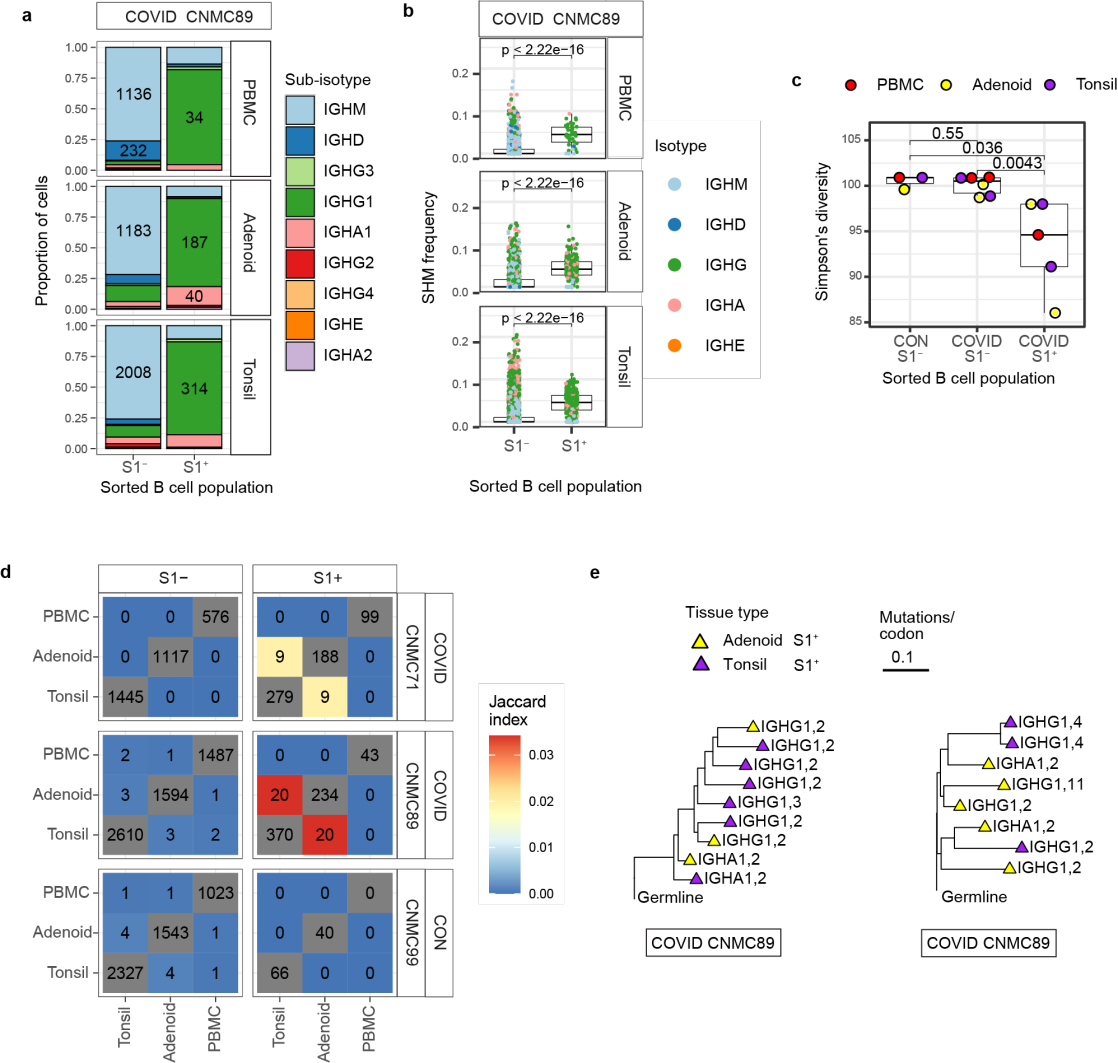
Single cell BCR sequencing of SARS-CoV-2 antigen-specific B cells a. Sub-isotype frequencies among S1^+^ and S1^−^ B cells from PBMC, adenoid, and tonsil of one COVID-19 convalescent donor (CNMC 89). Labels show the raw number of cells with a given sub-isotype and are only included for sub-isotypes that make up at least 10% of a given category. b. Somatic hypermutation (SHM) frequency among S1^+^ and S1^−^ B cells from PBMC, adenoid, and tonsil of CNMC 89. Mutation frequency calculated in V gene. c. Simpson’s diversity of S1^+^ and S1^−^ B cells from PBMCs, adenoids, and tonsils from 2 COVID-19 convalescent donors (COVID, CNMC 71 and 89) and one control (CON, CNMC 99). Lower Simpson’s diversity values indicate a greater frequency of large clones. To adjust for sequence depth, diversity is calculated as the mean of 1000 uniform resampling repetitions. d. Overlap of B cell clones among PBMC, tonsil, and adenoid from COVID and CON. Off-diagonal elements are colored by the Jaccard index of clonal overlap between the two tissues and are labelled by the raw number of overlapping clones. Diagonal elements are labelled by the total number of clones within a particular tissue. e. Clonal lineage trees from two of the largest S1^+^ B cell clones shared between tonsil and adenoid from CNMC 89. Triangles indicate S1^+^ cells, and tip color indicates tissue of origin. Isotype and CITE-seq cluster of each cell are listed next to the symbol. Branch lengths represent SHM frequency/codon in VDJ sequence according to the scale bar. Significance calculated with Mann Whitney U test.

BCR sequence analysis confirmed that S1^+^ B cells were primarily IgG1 and IgA1 class-switched cells (Fig. 3a, Extended Data Fig. 2d), with high frequencies of somatic hypermutation (SHM) in V_H_ genes (Fig. 3b, Extended Data Fig. 2e) and low clonal diversity compared to S1^−^ B cells, indicative of antigen-driven clonal expansion (Fig. 3c). The high mutation frequency in S1^+^ B cells is consistent with prior work showing that subjects with mild COVID-19 had higher frequencies of hypermutated memory B cells compared to those with severe COVID-19^22^ and suggests that these SARS-CoV-2-specific clones underwent somatic hypermutation in GCs.

Intriguingly, we also observed that a portion of S1^+^ B cell clones (a total of 83 cells from 29 clones: 20 clones from donor 89 and 9 from donor 71) were present in both the tonsils and adenoids (Fig. 3d). The shared S1^+^ clones were nearly all isotype-switched cells (Extended Data Fig. 2f) and, like the total S1^+^ B cell population, were comprised primarily of cells from cluster 2 (CD27^+^ memory B cells) (Fig. 2e). However, a small number of cells from shared clones in the tonsil of one donor were GC B cells (cluster 4) (Fig. 2e; Supplementary Table 6). The distribution of these shared clones across adenoid and tonsil within some clonal lineage trees suggested that B cell clones migrated between these oropharyngeal lymphoid tissues and raised the possibility that class switching can occur before, during, or after SHM (Fig. 3e). Thus, multimodal single cell analysis of the SARS-CoV-2-specific B cells both supports their emergence from GCs and suggests sharing and potential migration of clonally expanded B cells between oropharyngeal lymphoid tissues.

## Expanded GC populations after COVID-19

To determine whether prior SARS-CoV-2 infection can broadly alter the immune landscape of mucosal tissues beyond acute infection, we compared the immune cell profiles of tonsils, adenoids, and peripheral blood from individuals with a history of COVID-19 to those without, using both unsupervised analyses and manual gating of high-dimensional flow cytometry data (samples included in each analysis are listed in Supplementary Table 2). To probe cell populations in greater detail, CD19^+^ B, CD4^+^ T, and CD8^+^ T lymphocytes were first gated and then analyzed separately. Adenoids and tonsils were evaluated together, whereas PBMCs were examined on their own, to account for and increase sensitivity for detecting distinct populations in tissues and peripheral blood.

**Extended Data Figure 4.**
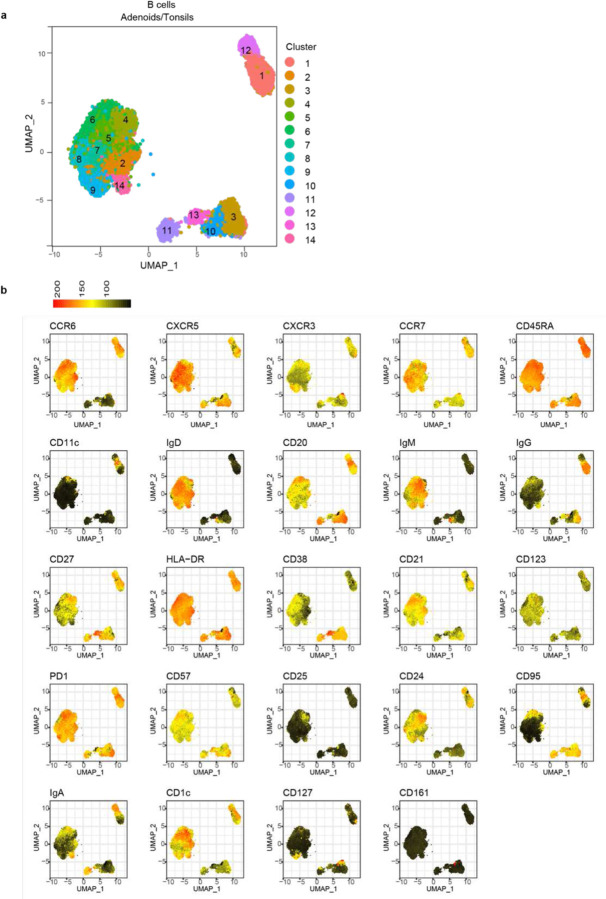
UMAP of unsupervised clustering of B cells from tonsil and adenoid a. Uniform manifold approximation and projection (UMAP) of unsupervised clustering of surface markers from flow cytometric analysis of CD19^+^ B cells from adenoid and tonsil. b. Heatmaps of marker/antibody expression overlayed on UMAP.

**Figure 4.**
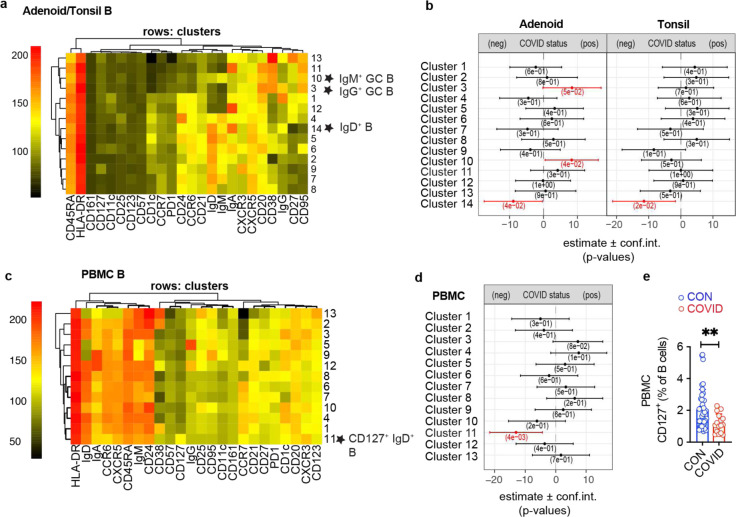
GC B cells are expanded in adenoids after COVID-19 a and c. Unsupervised clustering of CD19^+^ B cells from adenoid and tonsil (a) and PBMC (c) according to flow cytometric surface markers. Stars indicate clusters with significant differences (p<0.05) in COVID-19-convalescent samples (COVID) vs. controls (CON) (COVID adenoid n = 11, CON adenoid n = 33, COVID tonsil n = 15, CON tonsil n = 42, COVID PBMC n = 14, CON PBMC n = 36). b and d. Quantification of the effect of prior SARS-CoV-2 infection on CD19^+^ B cell clusters in adenoid and tonsil (b) and PBMC (d) estimated with a linear model controlling for age and sex. Regression coefficients with 95% confidence intervals and p values are shown. Significantly different clusters are highlighted in red. Analyzed samples are listed in Supplementary Table 2. Statistical analysis described in Methods. e. Frequency of CD127^+^ B cells in PBMC of COVID (n = 16) vs. CON (n = 41). Significance calculated using Mann-Whitney U test. Each symbol represents data from one donor. Mean ± S.D. are displayed. ** p<0.01.

In the unsupervised analysis of B cell phenotypes, we compared those with prior COVID-19 to control subjects while controlling for age and sex. This analysis highlighted 14 clusters and revealed more pronounced changes in the adenoids post-COVID-19 (Fig. 4a–b, Extended Data Fig. 4). Clusters 3 and 10 were significantly increased in the adenoids of participants with a history of COVID-19 (Fig. 4b); these clusters represented IgG^+^ and IgM^+^ GC B cells, respectively. In addition, cluster 14, which clustered with naïve B cells, was decreased in both adenoids and tonsils of COVID-19 convalescent subjects (Fig. 4a–b). In the peripheral blood, a CD127^+^IgD^+^ B cell cluster was also decreased following COVID-19 (Fig. 4c–d, Supplementary Fig. 4a–b); this was confirmed by manual gating on CD127^+^ B cells (Fig. 4e). Thus, prior COVID-19 is associated with prolonged changes in B cell populations well into convalescence, including persistent enrichment of GC B cells in the adenoids.

## Expanded Tfh populations after COVID-19

**Extended Data Figure 5.**
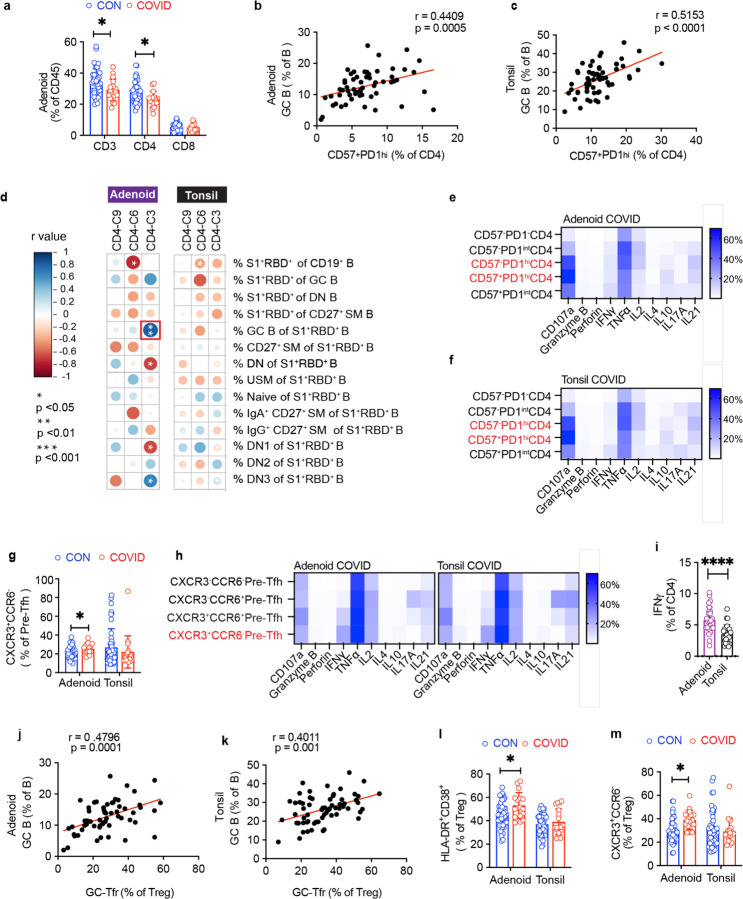
Phenotyping of expanded CD4^+^ T cell populations in tissue a. Comparison of CD3^+^, CD4^+^, and CD8^+^ T cell frequency in adenoid of COVID-19-convalescent donors (COVID) vs. controls (CON). b and c. Correlation between frequency of CD57^+^PD-1^hi^ CD4^+^ T cells and frequency of GC B in adenoids (b) and tonsil (c) (adenoid n = 59, tonsil n = 64, includes both COVID-19-convalescent samples and controls). d. Summary of correlations among various subsets of SARS-CoV-2 antigen-specific B cells and significantly different clusters from unsupervised analysis of tissue CD4^+^ T cells (clusters 3, 6, 9). USM = unswitched memory B, SM = switched memory B, DN = double negative B, GC = germinal center. e and f. Intracellular cytokine and cytotoxic factor expression in various CD4^+^ T cell subsets gated on CD57 and PD-1 from COVID-19-convalescent adenoids (e, n = 13) and tonsil (f, n = 13) after PMA and ionomycin stimulation. Mean frequency expressing each cytokine is plotted in the heatmap. g. Frequency of CXCR3^+^CCR6^−^ cells among pre-Tfh cells (PD-1^int^CXCR5^+^ conventional CD4^+^ T) in adenoids and tonsils of COVID vs. CON. h. Intracellular cytokine and cytotoxic factor expression in different pre-Tfh cell subsets gated on CXCR3 and CCR6 from COVID-19-convalescent adenoids (n = 13) and tonsils (n = 13) after PMA and ionomycin stimulation. Mean frequency expressing of each cytokine is plotted in the heatmap. i. Comparison of IFN-γ production by CD4^+^ T cells in adenoid versus tonsil following PMA/ionomycin stimulation (n = 26 which includes 13 COVID and 13 CON of each tissue). j. Correlation between frequency of GC-Tfr and GC B frequencies in adenoid (n = 59, includes both COVID and CON). k. Correlation between frequency of GC-Tfr and GC B frequencies in tonsil (n = 64, includes both COVID and CON). l and m. Frequencies of HLA-DR^+^CD38^+^ (d) CXCR3^+^CCR6^−^ (e) cells among Treg cells in adenoid and tonsil. (COVID adenoid n = 17, CON adenoid n = 42, COVID tonsil n = 18, CON tonsil n = 46). Gating strategy shown in Supplementary Fig. 5. Samples analyzed in panels a-c, g and j-m are listed in Supplementary Table 2 (COVID adenoid n = 17, CON adenoid n = 42, COVID tonsil n = 18, CON tonsil n = 46). Samples analyzed for panel d-e and h-i are in Supplementary Table 9. Each symbol represents data from one donor. Means ± S.D. are displayed on scatter and bar plots. Significance calculated using Mann-Whitney U test to compare two groups and Spearman’s rank test for correlations. * p<0.05

**Extended Data Figure 6.**
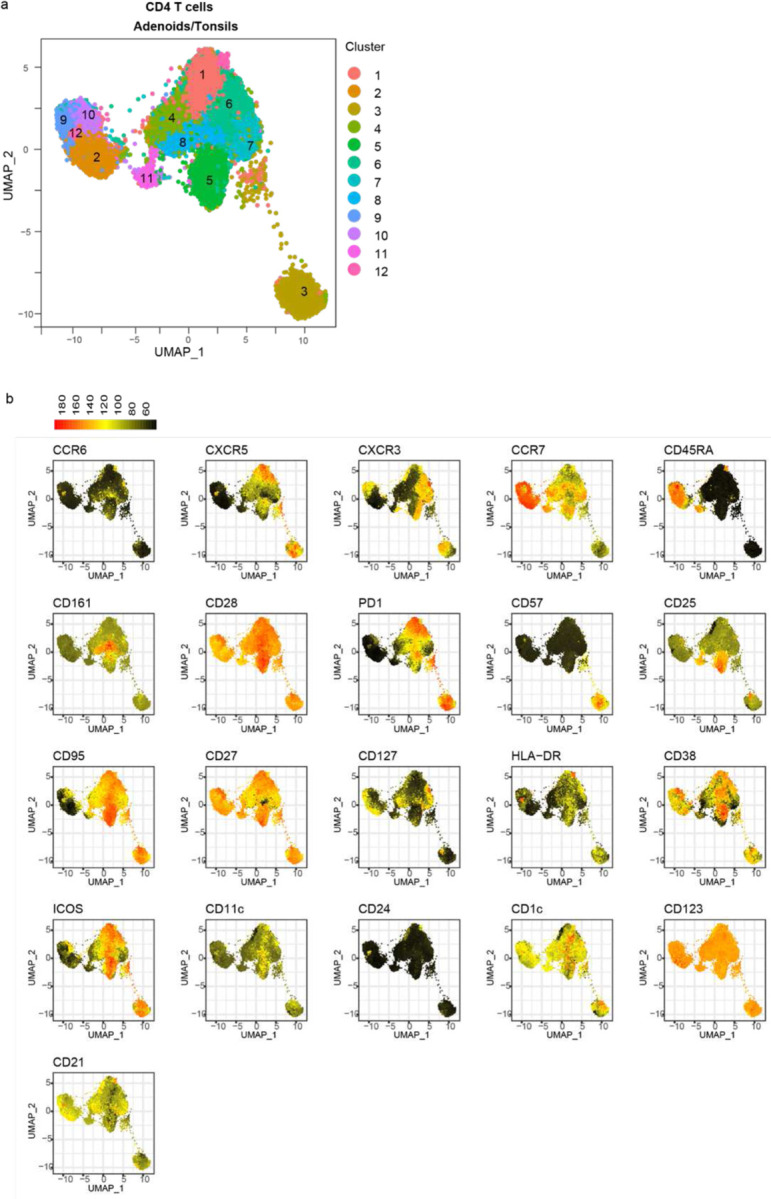
UMAP of unsupervised clustering of CD4^+^ T cells from tonsil and adenoid a. Uniform manifold approximation and projection (UMAP) of unsupervised clustering of surface markers from flow cytometric analysis of CD4^+^ T cells from adenoid and tonsil. b. Heatmaps of marker/antibody expression overlayed on UMAP.

**Figure 5.**
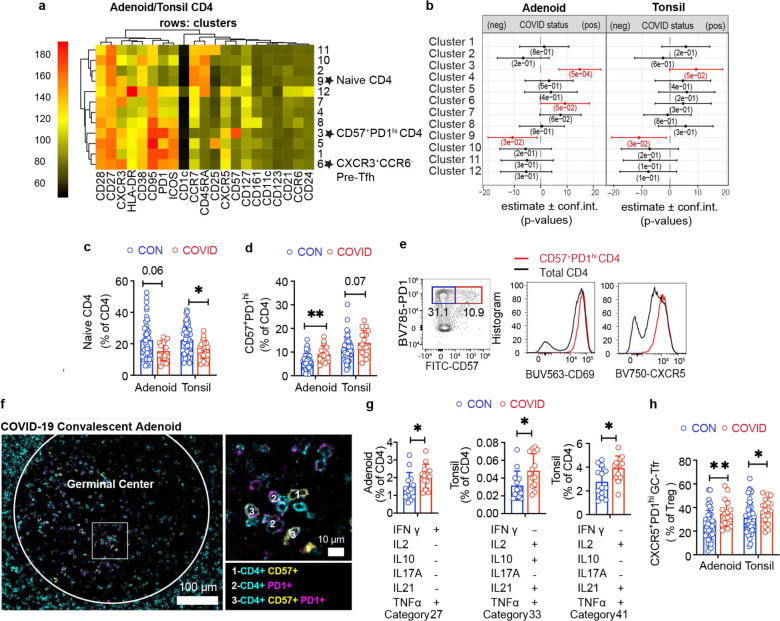
CD4^+^ Tfh cells are expanded in pharyngeal tissues post-COVID-19 a. Unsupervised clustering of CD4^+^ T cells from adenoid and tonsil according to surface markers from flow cytometry. Stars indicate clusters with significant differences (p<0.05) in COVID-19-convalescent samples (COVID) vs. controls (CON) (COVID adenoid n = 12, CON adenoid n = 38, COVID tonsil n = 15, CON tonsil n = 43). b. Quantification of the effect of prior SARS-CoV-2 infection on CD4^+^ T cell clusters in adenoid and tonsil estimated with a linear model controlling for age and sex. Regression coefficients with 95% confidence intervals and p values are shown. Significantly different clusters are highlighted in red. Statistical analysis is described in Methods. c. and d. Frequencies of naïve (CD45RA^+^CCR7^+^) CD4^+^ T cells (c) and CD57^+^PD-1^hi^ CD4^+^ T cells (d) in adenoids and tonsils of COVID vs. CON. e. Representative plots of CD69 and CXCR5 expression on CD57^+^PD-1^hi^ CD4^+^ T cells vs. total CD4^+^ T cells from tonsil. Phenotypes are similar in adenoid. f. Representative multicolor immunofluorescence staining of COVID adenoid showing the location of CD57^+^PD-1^hi^ CD4^+^ T cells in the GC. CD4 shown in cyan, CD57 in yellow, and PD-1 in magenta. GC boundaries were defined using Ki-67 (not shown) as demonstrated in Figure 1h. g. Significantly different cytokine combinations produced by tonsillar and adenoid CD4^+^ T cells from COVID (n = 13) vs. CON (n = 13) following PMA and ionomycin stimulation. Fifty-nine combinations of cytokines (IFN-γ, IL-2, IL-10, IL-17A, IL-21, and TNF-α) made by CD4^+^ T cells were included in the SPICE analysis (see Supplementary Fig. 6). h. Frequencies of CXCR5^+^PD-1^hi^ GC-Tfr cells in adenoid and tonsil of COVID vs. CON. Gating strategy shown in Supplementary Figure 5. Samples analyzed in panels c-d and h are listed in Supplementary Table 2 (COVID adenoid n = 17, CON adenoid n = 42, COVID tonsil n = 18, CON tonsil n = 46). Samples included in panel f-g are listed in Supplementary Table 9. Each symbol represents data from one donor. Means ± S.D. are displayed on scatter and bar plots. Significance calculated using Mann-Whitney U test. * p<0.05, ** p<0.01.

Post-COVID-19, we observed that the adenoids had lower percentages of CD4^+^ T cells (Extended Data Fig. 5a). Unsupervised clustering further underscored differences in COVID-19-convalescent samples (Fig. 5a–b, Extended Data Fig. 6a–b) that included a reduction in cluster 9, which represents naïve CD4^+^ T cells (CD45RA^+^CCR7^+^), in both tonsils and adenoids from COVID-19 convalescent subjects (Fig. 5a–b). Traditional gating confirmed decreased percentages of naïve CD4^+^ T cells in lymphoid tissue (Fig. 5c).

Conversely, cluster 3, which represents a CD57^+^PD-1^hi^ subset, was significantly enriched after COVID-19 in both the adenoids and tonsils (Fig. 5a–b); manual gating confirmed an expanded CD57^+^PD-1^hi^ CD4^+^ T cell population in the tissues (Fig. 5d). CD57 has been described as a marker of T cell senescence but is also found on a population of tonsillar GC-Tfh cells^23–25^, a subset of CD4^+^ T helper cells that provide contact-mediated signals to antigen-stimulated B cells for GC formation and maintenance. Compared to the total CD4^+^ T cell population in the tissues, the CD57^+^PD-1^hi^ CD4^+^ T cell population exhibited higher expression of CXCR5 and CD69, indicative of a Tfh phenotype and characteristic of tissue-resident memory T (TRM) cells, respectively^6^ (Fig. 5e). Imaging studies revealed that CD57^+^PD-1^hi^ CD4^+^ T cells were located within the GC (Fig. 5f). Moreover, their frequency positively correlated with the proportion of GC B cells in both the adenoids and tonsils (Extended Data Fig. 5b–c). The percentage of cluster 3 also positively correlated with the percentage of S1^+^RBD^+^ B cells that were GC B cells in the adenoids (Extended Data Fig. 5d), supporting the idea that these T cells contributed to the generation and persistence of SARS-CoV-2-specific GC responses. Consistent with these data, stimulation with PMA and ionomycin showed that CD57^+^PD-1^hi^ CD4^+^ T cells from the adenoids and tonsils produced IL-21 and IL-10, cytokines that facilitate GC formation and B cell antibody secretion (Extended Data Fig. 5e–f).

Cluster 6 was also significantly increased in COVID-19-convalescent subjects but only in the adenoids (Fig. 5a–b, Extended Data Fig. 5g). This cluster represented a pre-Tfh cell population (CD45RA^−^CXCR5^+^PD-1^int^) that expressed CXCR3 but not CCR6 (Extended Data Fig. 6a–b), a combination of markers associated with IFN-γ/Th1 cytokine production^26^. Upon PMA and ionomycin stimulation, a high percentage of CXCR3^+^CCR6^−^ pre-Tfh cells produced IFN-γ (Extended Data Fig. 5h), suggesting that type 1 (IFN-γ-associated) T cell responses were induced as part of the anti-viral response to SARS-CoV-2 in the adenoids. To further evaluate T cell function, we examined the overall patterns of cytokine production from tonsil and adenoid cells stimulated with PMA and ionomycin. Global evaluation of CD4^+^ T cell polyfunctionality by SPICE (Simplified Presentation of Incredibly Complex Evaluations) revealed several combinations of cytokines were significantly enriched in the post-COVID-19 group (Fig. 5g, Supplementary Fig. 6); two of these combinations included IL-21 (categories 33 and 41), suggesting production by Tfh cells. One of the enriched combinations included IL-10 in addition to IL-21; IL-10 production by Tfh cells is important for maintaining GCs in viral infections^27^ and is expressed by CD57+ Tfh cells. Notably, increased IFN-γ was also part of a cytokine pattern (category 27) specifically enriched in adenoids post-COVID-19, perhaps reflecting the increased CXCR3^+^CCR6^−^ pre-Tfh population (cluster 6) we observed. Consistent with this, we find more robust IFN-γ production by CD4^+^ T cells in adenoids compared to the tonsils indicating inherent differences in the T cell populations in these lymphoid tissues (Extended Data Fig. 5i).

Finally, in accordance with evidence of robust GC responses in the adenoids and tonsils post-COVID-19, we found more T follicular regulatory (Tfr) cells (CXCR5^+^PD-1^hi^) among CD127^−^CD25^+^ regulatory T cells in COVID-19-convalescent tonsils and adenoids (Fig. 5h); the frequency of these cells positively correlated with the percentage of GC B cells (Extended Data Fig. 5j–k). Similar to the characteristics we found in adenoid CD4^+^ T cells, regulatory T cells (CD25^+^CD127^−^) in the adenoids were also more activated after COVID-19, with a higher percentage of HLA-DR^+^CD38^+^ and CXCR3^+^CCR6^−^ cells, again suggesting the adenoids may have been primed by a stronger immune response to SARS-CoV-2 than the tonsils (Extended Data Fig. 5l–m). Thus, we find an expansion of percentages of Tfh as well as Tfr cells in the tonsils and adenoids that extends into convalescence, providing further evidence for prolonged GC responses to SARS-CoV-2 in the upper respiratory tract of children.

## Enrichment of activated circulating Tfh cells in the blood following COVID-19

**Extended Data Figure 7.**
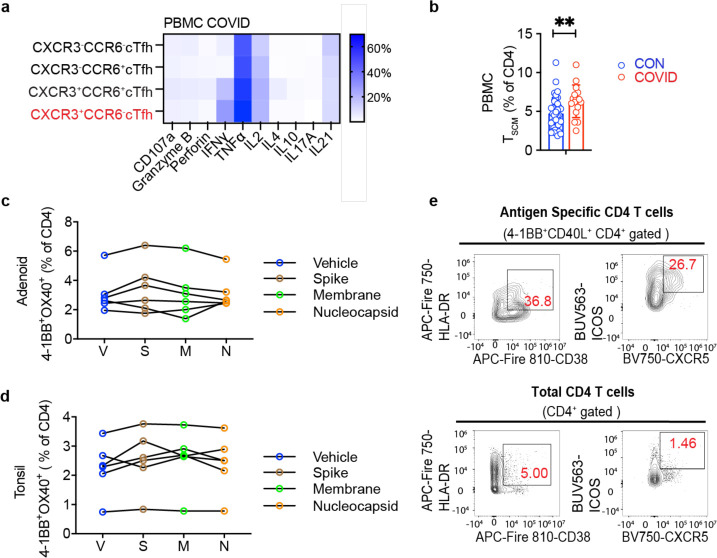
SARS-CoV-2 antigen-specific CD4^+^ T cells following COVID-19 a. Intracellular cytokine and cytotoxic factor production by various circulating Tfh (cTfh) cell subsets in PBMC gated by CXCR3 and CCR6 from COVID-19-convalescent donors (n = 4) following PMA and ionomycin stimulation. Mean frequency expressing each cytokine is plotted in heatmap. b. Frequency of stem cell-like memory CD4^+^ T (T_SCM_, CD45RA^+^CCR7^+^CD28^+^CD27^+^CD95^+^) subsets in PBMC of COVID-19-convalescent donors (COVID) vs. controls (CON) (COVID = 16, CON = 41). Significance calculated using Mann-Whitney U test. Gating strategy in Supplementary Fig. 7. c and d. Frequencies of AIM^+^ (OX40^+^4–1BB^+^) CD4^+^ T cells from adenoid (c) and tonsil (d) of COVID-19-convalescent donors following SARS-CoV-2 peptide pool stimulation (adenoid n = 6, tonsil n = 6). Significance calculated with Wilcoxon signed rank test for paired samples from the same donor. e. Flow cytometry plots showing frequency of HLA-DR^+^CD38^+^ and ICOS^+^CXCR5^+^ cells from concatenated antigen-specific CD4^+^ T cells from PBMC following SARS-CoV-2 peptide stimulation compared to total CD4^+^ T cells. AIM^+^ CD4^+^ T cells were concatenated from all three peptide pool stimulations of PBMCs from all 6 donors. Samples analyzed in panel a, c, and d are listed in Supplementary Table 9, and in panel b are in Supplementary Table 2. ** p<0.01.

**Figure 6.**
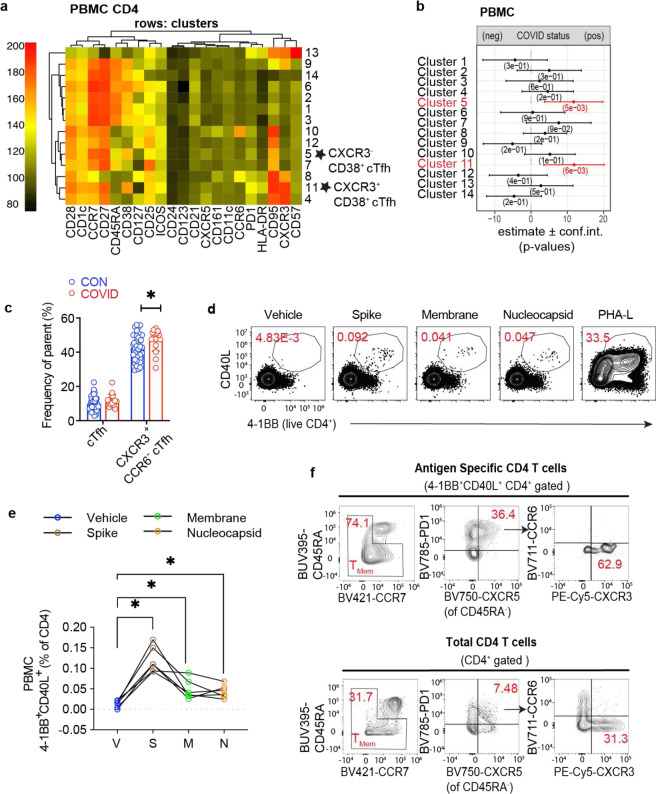
SARS-CoV-2 antigen-specific CD4^+^ T cells in PBMC following COVID-19 a. Unsupervised clustering of CD4^+^ T cells from PBMC according to surface markers from flow cytometric analysis. Stars indicate clusters with significant differences (p<0.05) in COVID-19-convalescent samples (COVID) vs. controls (CON) (COVID n = 13, CON n = 34). b. Quantification of the effect of prior SARS-CoV-2 infection on CD4^+^ T cell clusters in PBMC estimated with a linear model controlling for age and sex. Regression coefficients with 95% confidence intervals and p values are shown. Significantly different clusters are highlighted in red. Statistical analysis is described in Methods. c. Frequencies of cTfh (CD45RA^−^CXCR5^+^PD-1^+^) and CXCR3^+^CCR6^−^ cTfh cells in PBMC of COVID (n=16) vs. CON (n=41). Significance calculated with Mann-Whitney U. d. Representative flow cytometry plots showing gating of antigen-specific CD4^+^ T cells from PBMC of a COVID-19-convalescent donor expressing activation induced markers (AIM^+^: CD40L^+^4–1BB^+^) following stimulation with SARS-CoV-2 peptide pools of spike, membrane, and nucleocapsid. DMSO was used as negative control (vehicle), and PHA-L was used as positive control. e. Frequencies of AIM^+^ CD4^+^ T cells from PBMC of COVID-19-convalescent donors following SARS-CoV-2 peptide pool stimulation (n = 6). Significance calculated with Wilcoxon signed rank test for paired samples from the same donor. f. Flow cytometry plots showing frequency of T_Mem_, cTfh (CD45RA^−^CXCR5^+^PD-1^+^), and CXCR3^+^CCR6^−^ cTfh cells from concatenated antigen-specific CD4^+^ T cells following SARS-CoV-2 peptide stimulation compared to total CD4^+^ T cells in PBMC. AIM^+^ CD4^+^ T cells were concatenated from all three peptide pool stimulations from all 6 donors. Gating strategy shown in Supplementary Figure 8. Samples used in AIM analyses are shown in Supplementary Table 9. * p<0.05.

Because lymphocyte populations in the peripheral blood differ from the tonsil and adenoid, we evaluated PBMCs separately; unsupervised grouping of high-dimensional flow cytometry data revealed two clusters (cluster 5 and cluster 11) that were increased following COVID-19 (Fig. 6a–b, Supplementary Fig. 7a–b); both contained circulating Tfh (cTfh)-like cells (CD45RA^−^CXCR5^+^PD-1^+^) that expressed CD38, a marker of recently activated T cells^28^; cluster 11 was CXCR3^+^ while cluster 5 was not. Although we did not find increased percentages of total cTfh cells by manual gating, we found that cTfh cells were skewed to a CXCR3^+^CCR6^−^ phenotype in the COVID-19-experienced group (Fig. 6c); these cells produced IFN-γ upon stimulation with PMA and ionomycin (Extended Data Fig. 7a). Analogous to prior reports, we also observed an increased frequency of stem cell-like memory CD4^+^ T (T_SCM_) (CD45RA^+^CCR7^+^CD28^+^CD27^+^CD95^+^) (Extended Data Fig. 7b), perhaps reflecting long-lived memory T cells following recovery from COVID-19 in children^29^.

To identify SARS-CoV-2 antigen-specific CD4^+^ T cells, we stimulated tonsil, adenoid, and peripheral blood mononuclear cells with spike (S), membrane (M), and nucleocapsid (N) peptide pools and assessed the activation-induced markers (AIM) CD40L, OX40, and 4–1BB on T cells. Although we were not able to precisely identify and phenotype the SARS-CoV-2-specific T cells in the adenoids and tonsils due to the highly activated status of T cells at baseline without stimulation in these tissues (Extended Data Fig. 7c–d), SARS-CoV-2-reactive CD4^+^ T cells were identified in the peripheral blood with the greatest responses to the S peptide pool (Fig. 6d–e). By concatenating all the peptide-activated CD4^+^ T cells, we found that the SARS-CoV-2-responsive CD4^+^ T cells in the peripheral blood were primarily memory cells that were enriched for CXCR3^+^ cTfh cells (CD45RA^−^CXCR5^+^PD-1^+^) and expressed high levels of HLA-DR, CD38, and ICOS (Fig. 6f, Extended Data Fig. 7e). This enrichment of CXCR3^+^ cTfh-like cells in the peripheral blood, a portion of which are SARS-CoV-2-specific, likely parallels the skewing of pre-Tfh cells we found in the pharyngeal lymphoid tissue in response to SARS-CoV-2.

## Expanded tissue resident CD8^+^ cells after COVID-19

**Extended Data Figure 8.**
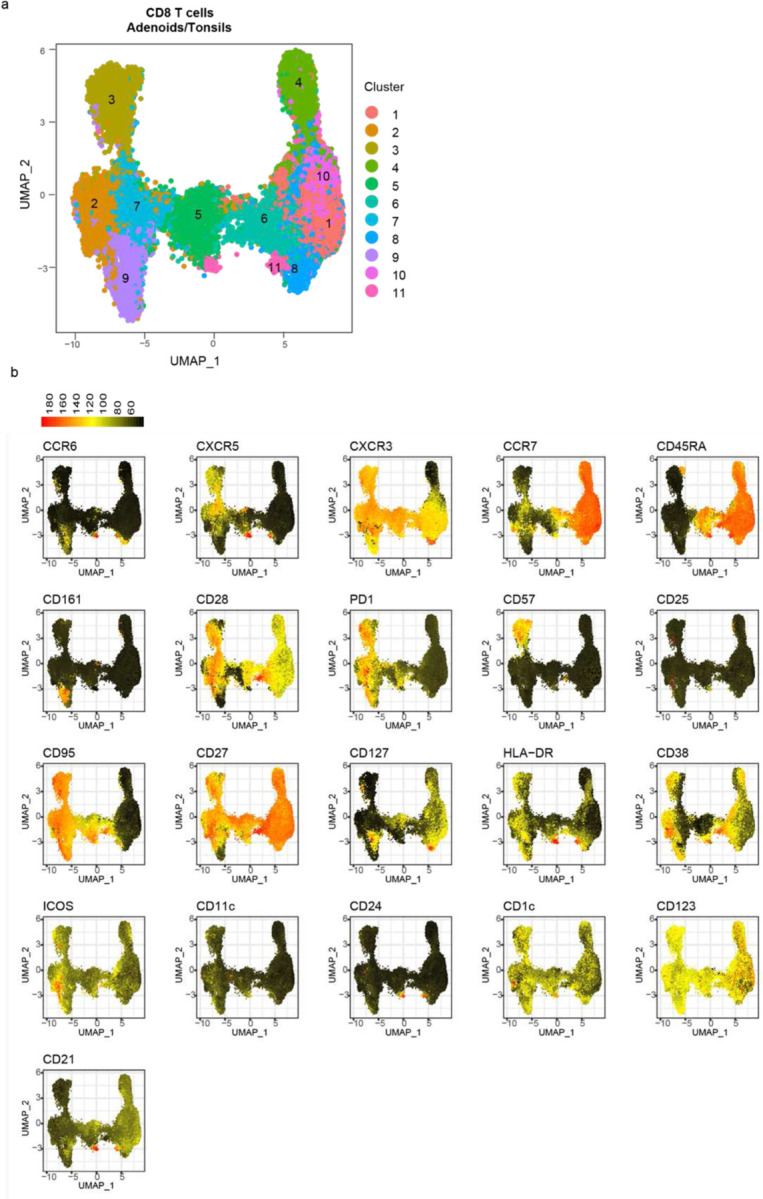
UMAP of unsupervised clustering of CD8^+^ T cells from tonsil and adenoid a. Uniform manifold approximation and projection (UMAP) of unsupervised clustering of surface markers from flow cytometric analysis of CD8^+^ T cells from adenoid and tonsil. b. Heatmaps of marker/antibody expression overlayed on UMAP.

**Extended Data Figure 9.**
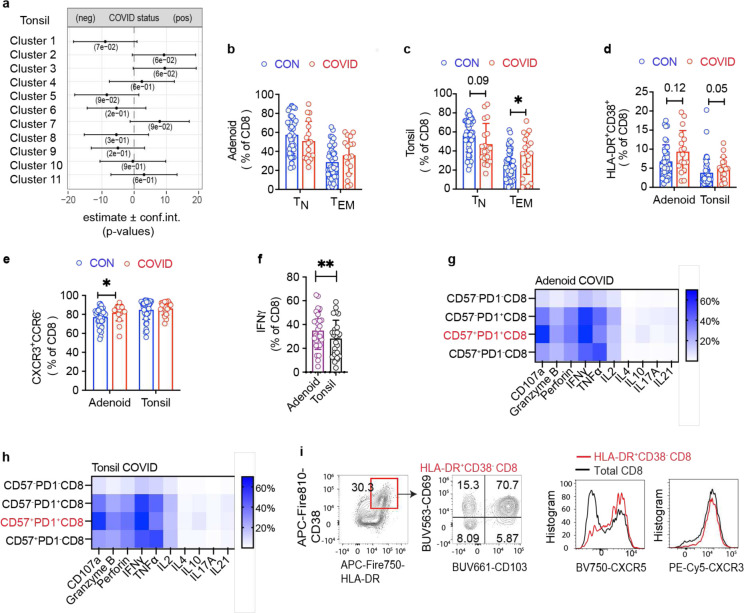
Phenotyping of CD8^+^ T cells from tonsil and adenoid a. Quantification of the effect of prior SARS-CoV-2 infection on CD8^+^ T cell clusters in tonsil estimated with a linear model controlling for age and sex. Regression coefficients with 95% confidence intervals and p values are shown (COVID n = 15, CON n = 42). b and c. Frequencies of naïve (T_N_, CD45RA^+^CCR7^+^) and effector memory (T_EM_, CD45RA^−^CCR7^−^) CD8^+^ T cells in adenoid (b) and tonsil (c) of COVID-19-convalescent samples (COVID) vs. controls (CON). d and e. Frequency of HLA-DR^+^CD38^+^ (d) and CXCR3^+^CCR6^−^ (e) cells among CD8^+^ T cells in adenoid and tonsil from COVID vs. CON. f. Comparison of IFN-γ production by CD8^+^ T cells in adenoid versus tonsil following PMA and ionomycin stimulation (n = 26 which includes 13 COVID and 13 CON of each tissue). g and h. Intracellular cytokine and cytotoxic factor production by different CD8^+^ T cell subsets gated by CD57 and PD-1 from adenoid (g, n = 13) and tonsil (h, n = 13) from COVID-19-convalescent donors. Mean expression of each cytokine is plotted in the heatmap. i. Representative flow cytometry plots showing the expression of CD69, CD103, CXCR3, and CXCR5 levels on HLA-DR^+^CD38^+^ CD8^+^ T cells in tonsil. Phenotypes are similar in adenoid. Gating strategy shown in Supplementary Fig. 5. Samples analyzed in panels a-e are listed in Supplementary Table 2 (COVID adenoid n = 17, CON adenoid n = 42, COVID tonsil n = 18, CON tonsil n = 46), and in panel f-h are in Supplementary Table 9. Each symbol represents data from one donor. Means ± S.D. are displayed on scatter and bar plots. Significance calculated using Mann-Whitney U test. * p<0.05, ** p<0.01.

**Figure 7.**
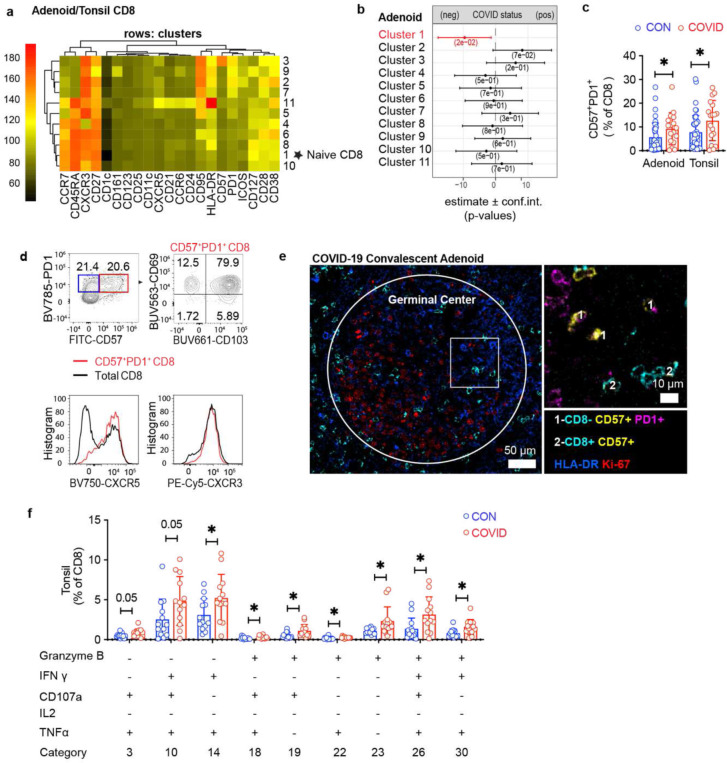
Tissue-resident memory CD8^+^ T cells are expanded in pharyngeal tissues post-COVID-19 a. Unsupervised clustering of CD8^+^ T cells from adenoid and tonsil according to surface antibody expression from flow cytometry analysis. Stars indicate clusters with significant differences (p<0.05) in COVID-19-convalescent samples (COVID) vs. controls (CON). (COVID adenoid n = 12, CON adenoid n = 35, COVID tonsil n = 15, CON tonsil n = 42). b. Quantification of the effect of prior SARS-CoV-2 infection on CD8^+^ T cell clusters in adenoid estimated with a linear model controlling for age and sex. Regression coefficients with 95% confidence intervals and p values are shown. Significantly different clusters are highlighted in red. Statistical analysis is described in Methods. c. Frequency of CD57^+^PD-1^+^ CD8^+^ T cells in adenoid and tonsil from COVID vs. CON (COVID adenoid n = 17, CON adenoid n = 42, COVID tonsil n = 18, CON tonsil n = 46). d. Representative flow cytometry plots showing CD69, CD103, CXCR5, and CXCR3 expression on CD57^+^PD-1^+^ CD8^+^ T cells from tonsil. Phenotypes are similar in adenoid. e. Representative multicolor immunofluorescence staining of adenoid from COVID-19-convalescent donor showing the location of CD57^+^PD-1^+^ CD8^+^ T in the GC. CD8 is shown in cyan, CD57 is yellow, PD-1 is pink. HLA-DR (blue) stains follicles, and Ki-67 (red) stains GC. f. Significantly different cytokine combinations produced by tonsillar CD8^+^ T cells from COVID (n=13) vs. CON (n=13) following PMA and ionomycin stimulation. Thirty-one combinations of cytotoxic factors and cytokines (granzyme B, IFN-γ, CD107a, IL-2 and TNF-α) made by CD8^+^ T cells were included in the SPICE analysis (see Supplementary Fig. 9). Gating strategy shown in Supplementary Figure 5. Samples analyzed in panels a-c are listed in Supplementary Table 2. Samples included in panels e-f are listed in Supplementary Table 9. Each symbol represents data from one donor. Means ± S.D. are displayed in scatter and bar plots. Significance calculated using Mann-Whitney U test. * p<0.05.

To further evaluate anti-viral responses, we examined CD8^+^ T cell in the tonsils and adenoids. With unsupervised clustering, we found that cluster 1, which represented naïve CD8^+^ T cells, decreased following COVID-19 in the adenoids (Fig. 7a–b, Extended Data Fig. 8a–b; Extended Data Fig. 9a); manual gating revealed a similar, but not significant trend in both adenoids and tonsils, in addition to more effector memory CD8^+^ T cells in the tonsils of the COVID-19 experienced cohort (Extended Data Fig. 9b–c). Although not statistically significant, both adenoids and tonsils exhibited increases in cluster 2 and cluster 3 following COVID-19. These two clusters represented activated effector memory CD8^+^ T cells (HLA-DR^+^CD38^+^CXCR3^+^CCR7^−^CD45RA^−^); cluster 2 expressed higher CD38, while cluster 3 expressed more CD57. Manual gating demonstrated that CD57^+^PD-1^+^ CD8^+^ T cells were significantly higher in adenoids and tonsils (Fig. 7c), while activated HLA-DR^+^CD38^+^ CD8^+^ T cells trended higher in tonsils of the COVID-19-convalescent group (Extended Data Fig. 9d). As in CD4^+^ T cells, the COVID-19-convalescent adenoids also had significantly more CXCR3^+^CCR6^−^ CD8^+^ T cells (Tc1 skewed) (Extended Data Fig. 9e). Furthermore, CD8^+^ T cells in the adenoid produced more IFN-γ than those in the tonsils upon PMA/ionomycin stimulation, again indicating the ability of the adenoids to create a more IFN-γ rich environment during the anti-viral response (Extended Data Fig. 9f).

CD8^+^ T cells expressing the senescence marker CD57 and inhibitory surface protein PD-1 are expanded in the peripheral blood of adults with moderate and severe COVID-19; however, the function of these cells and whether they represent a non-functional “exhausted” population is not clear^30,31^. We found that CD57^+^PD-1^+^ CD8^+^ T cells in the adenoids and tonsils had robust pro-inflammatory cytokine and cytotoxic factor production following PMA and ionomycin stimulation (Extended Data Fig. 9g–h).

Further analysis of both CD57^+^PD-1^+^CD8^+^ T cells in the tissue showed that the vast majority expressed the tissue-resident markers CD103 and CD69 in addition to CXCR5 (Fig. 7d) and these cells were found in the GC (Fig. 7e). HLA-DR^+^CD38^+^ CD8^+^ T cells also expressed CD103, CD69, and CXCR5 (Extended Data Fig. 9i). CXCR5^+^CD8^+^ T cells in lymphoid tissue have been shown to resemble stem-like or progenitor cells that maintain anti-viral function in chronic viral infections^32–37^. The role of these cells in the cellular immune response to an acute respiratory virus like SARS-CoV-2 is unknown, but their expansion and location in the GC raises questions about their roles in GCs and other anti-viral responses. In line with the expansion of activated CD8^+^ T cells in these tissues, evaluation of global cytokine production from CD8^+^ T cells by SPICE revealed multiple combinations of cytokines and cytotoxic molecules were significantly enriched post-COVID-19, most notably in the tonsils (Fig. 7f, Supplementary Fig. 9). Thus, activated CD8^+^ T cell populations were enriched in the pharyngeal lymphoid tissues post-COVID-19.

**Extended Data Figure 10.**
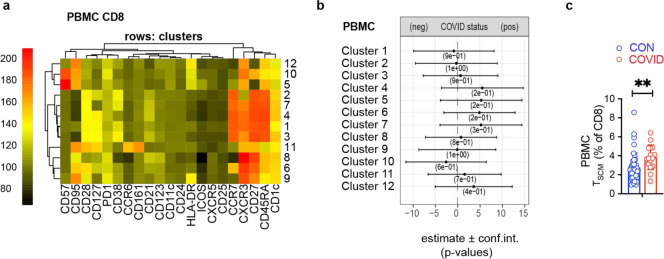
Phenotyping of CD8^+^ T cells from PBMC a. Unsupervised clustering of CD8^+^ T cells from PBMC according to surface antibodies from flow cytometric analysis. No clusters showed significant differences (p<0.05) in COVID-19-convalescent samples (COVID) vs. controls (CON) (COVID n = 13, CON n = 34). b. Quantification of the effect of prior SARS-CoV-2 infection on CD8^+^ T cell clusters in PBMC estimated with a linear model controlling for age and sex. Regression coefficients with 95% confidence intervals and p values are shown. So significantly different clusters were found. Statistical analysis is described in Methods. c. Frequency of T stem cell-like memory (T_SCM_, CD45RA^+^CCR7^+^CD28^+^CD27^+^CD95^+^) among CD8^+^ T cells in PBMC of COVID (n = 16) vs CON (n = 41). Gating strategy shown in Supplementary Fig. 8. Means ± S.D. are displayed on scatter and bar plots. Significance calculated using Mann-Whitney U test. Samples analyzed are listed in Supplementary Table 2. Each symbol represents data from one donor. ** p<0.01

In contrast, we did not find significant differences in CD8^+^ T cells in the PBMCs (Extended Data Fig. 10a–b, Supplementary Fig. 10a–b), with the exception of more abundant CD8^+^ T_SCM_ cells (CD45RA^+^CCR7^+^CD28^+^CD27^+^CD95^+^) seen by manual gating of COVID-19-convalescent samples (Extended Data Fig. 10c), parallel to our findings in peripheral CD4^+^ T cells and perhaps reflecting long-lived memory populations.

Together, these results provide evidence of activated and cytotoxic CD8^+^ TRM cells associated with increased cytokine production and GC localization in the pharyngeal lymphoid tissue, suggesting longer lasting effects of prior infection on these tissues compared to peripheral blood in convalescence. Thus, the pharyngeal lymphoid tissues may provide a unique window into the prolonged effects of SARS-CoV-2 infection.

## Viral RNA persistence in the pharyngeal tissue

**Figure 8.**
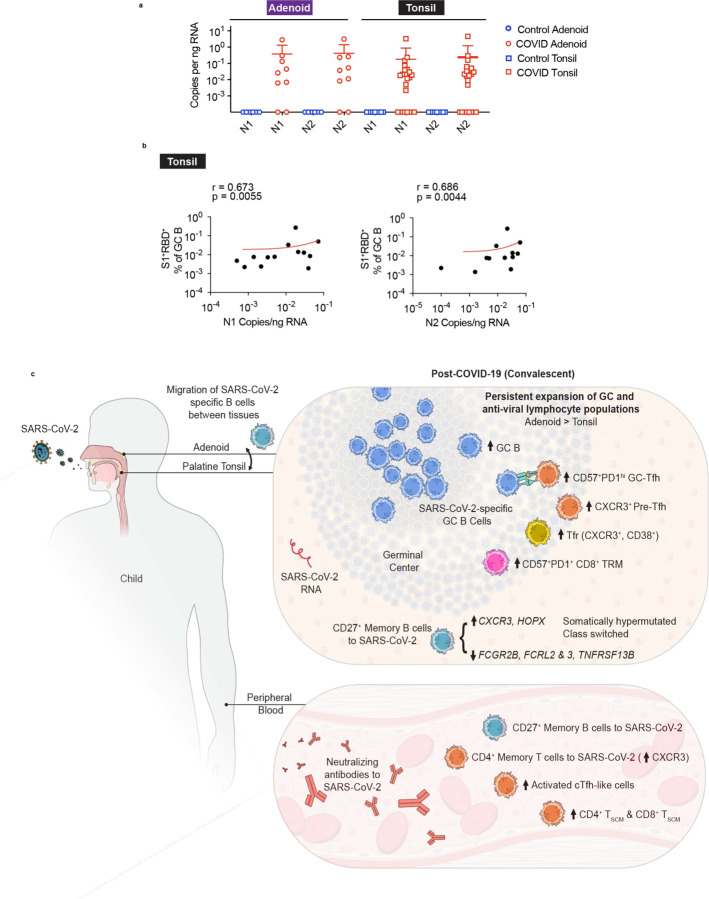
Persistence of SARS-CoV-2 RNA in the pharyngeal tissues post-COVID-19 a. Quantification of SARS-CoV-2 nucleocapsid RNA by digital droplet PCR (ddPCR) from adenoid and tonsil FFPE tissue blocks (COVID adenoid n = 9, control adenoid = 6, COVID tonsil n = 22, control tonsil n = 9). N1 and N2 represent two regions of the gene encoding the SARS-CoV-2 nucleocapsid. Each symbol represents data from one donor. Means ± S.D. are displayed. b. Correlation of nucleocapsid (N1 and N2) copies per nanogram RNA with percentage of S1^+^RBD^+^ B cells among GC B cells from tonsils following SARS-CoV-2 infection (COVID tonsil n = 13). c. Schematic illustrating the immunologic profile of the oropharyngeal lymphoid tissues and peripheral blood of COVID-19-convalescent children including (1) SARS-CoV-2-specific GC and memory B cells with overlapping clones in the tonsils and adenoids, (2) persistent changes in lymphocyte populations involved in germinal center and anti-viral responses, which were most prominent in the adenoid, with type 1 skewing of several T lymphocyte populations, and (3) persistence of viral RNA in the tissue. Samples analyzed are in Supplementary Table 7. Each symbol represents data from one donor. Means ± S.D. are displayed in scatter plots. Correlations assessed with Spearman’s rank correlation.

Given the apparent prolonged immune activation we observed in the pharyngeal tissue of children post-SARS-CoV-2 infection, we evaluated these tissues for evidence of viral persistence. RNA isolated from formalin-fixed, paraffin-embedded (FFPE) samples of tonsils and adenoids were analyzed by digital droplet PCR (ddPCR) for evidence of SARS-CoV-2 nucleocapsid RNA (N1 and N2). Viral RNA was found in multiple samples of COVID-19-convalescent tissues, despite negative PCRs from nasopharyngeal swabs at the time of surgery (Fig. 8a, Supplementary Table 7). SARS-CoV-2 was detected in 7 out of 9 FFPE adenoid blocks and 15 out of 22 FFPE tonsil blocks from COVID-19-convalescent individuals, but not in any control tissue samples. In several samples, participants’ previous positive PCR from a nasal swab was over 100 days prior to surgery, including one which was 303 days before surgery. Moreover, the copies of viral RNA significantly correlated with the percentage of S1^+^RBD^+^ cells among GC B cells in the tonsil (Fig. 8b). Although SARS-CoV-2 RNA was found in only a subset of post-COVID-19 tissues and we were unable to detect viral protein, these results raise the question of whether antigen persistence contributes to the prolonged lymphoid and GC responses we found following COVID-19.

## Discussion

Analysis of the tonsils and adenoids offers a unique opportunity to evaluate immune responses to a novel respiratory virus at the primary site of infection. Here, we demonstrate (1) direct evidence of robust SARS-CoV-2 antigen-specific GC and memory B cell responses in the upper respiratory tract lymphoid tissues post-COVID-19; (2) the presence of overlapping S1-reactive B cell clones in the tonsils and adenoids highlighting the dynamic nature of these anti-viral responses, (3) long-lasting expansion of cells involved in GC and anti-viral responses, including GC B, Tfh, Tfr, and effector CD8^+^ TRM cells in the lymphoid tissues and cTfh1 cells in the peripheral blood, (4) type 1 (IFN-γ-associated) skewing with CXCR3^+^ T lymphocytes, particularly in the adenoids, with corresponding changes in antigen-specific memory B cells, and (5) persistence of SARS-CoV-2 RNA in the lymphoid tissues months after infection. Together, these results demonstrate ongoing, tissue-specific immune responses to SARS-CoV-2 in oropharyngeal lymphoid tissues of children in the convalescent phase.

We identified 24 children out of 110 with evidence of prior COVID-19, most of whom were unaware of their infection status and/or asymptomatic. We note that these samples were taken prior to the availability of vaccination for children and thus reflected true infection. The high percentage of positive children during this period of collection, ending in early 2021, is notable and underscores the extent of infection present in this urban population^38,39^. In most of our pediatric participants, we found SARS-CoV-2-specific memory B cells in the peripheral blood and oropharyngeal tissue, indicating sustained humoral memory in both the local tissue and blood. Nonetheless, a few participants had low frequencies of memory B cells in the peripheral blood and/or oropharyngeal tissue and/or low serum neutralizing antibody titers, reflecting heterogeneous responses that may leave some children prone to repeat infection.

Other groups have noted lasting changes in the T and B cell populations in the peripheral blood of adults months after COVID-19^40–42^; a recent analysis of immune cells in the nasal mucosa also revealed enrichment of activated CD38^+^ CD8^+^ TRM and CD127^+^ granulocytes weeks after acute infection^43^. In our analysis of both upper respiratory tissue and peripheral blood, we see prominent changes in tonsils and adenoids compared to peripheral blood, providing evidence for tissue-specific anti-viral immune responses. Moreover, many of the enriched B and T cell populations we found in the pharyngeal tissue are functional tissue-resident populations involved in GC development and anti-viral responses that likely remain at the primary site of infection for months and even years, poised to provide localized immune protection^44^. These expanded tissue-resident T cell populations, including pre-Tfh and CD8^+^ TRM, exhibit type 1 skewing and may have created an IFN-γ-rich environment that led to upregulation of *CXCR3* and *HOPX* among S1^+^ B cells in the tissue. Strong local IFN (Type I and Type II) responses were recently found in the upper airways of infected children and likely led to enhanced viral control and milder disease compared to adults^45,46^. Here, we provide evidence for prolonged IFN-γ-induced responses in convalescent children. Whether immunization also generates immunity to SARS-CoV-2 in the upper respiratory tract and how this compares to natural infection are important questions that may have implications for determining optimal routes of vaccination.

Among the pharyngeal tissues we evaluated, the adenoids sustained more significant changes following COVID-19 than the tonsils; the frequency of SARS-CoV-2 specific B cells in the adenoids also correlated strongly with serum neutralizing titers for several variants including omicron providing evidence for the key role of this lymphoid tissue in anti-viral responses. Although they are both pharyngeal mucosal tissues, the adenoids and palatine tonsils differ in a number of ways. The adenoids are in the nasopharynx and have a respiratory epithelium, while the palatine tonsils are in the oropharynx and have a stratified squamous epithelium. We also found that T cells in the adenoid can produce more IFN-γ. These, as well as differences in the other immune cell populations, may make the adenoids more susceptible to immune activation during infection with a respiratory virus like SARS-CoV-2 than the tonsils. Although the adenoids are only one of many lymphoid structures in the upper respiratory tract with likely redundant function in generating an immune response to respiratory viruses, our data trigger questions as to whether adenoidectomy and tonsillectomy affect immune responses to SARS-CoV-2.

Longitudinal studies of SARS-CoV-2-specific B cells suggest continued maturation in GCs months after infection with peripheral memory B cell clones acquiring greater somatic hypermutation with time, possibly due to persistence of antigen in the tissue^17,41,47^. Maintenance of SARS-CoV-2-specific GC B cells and Tfh cells has also been noted in lung and lung-associated lymph nodes of 4 organ donors; in one case, this was observed at least 6 months following infection^13^. Using tissues from tonsillectomies and adenoidectomies, we were able to directly query lymphoid tissue at the primary site of infection in the upper respiratory tract and find compelling evidence of ongoing SARS-CoV-2-specific GC reactions with Tfh and effector memory CD8^+^ T cell expansion, perhaps in response to persistent viral antigen in the tissue months after the acute infection. Our results suggest these tissues can provide a powerful tool for examining responses to SARS-CoV-2.

Moreover, our observation of overlapping S1^+^ B cells clones between tonsil and adenoid tissue suggests that antigen-specific B cells may migrate between these tissues as part of an ongoing immune response. B cells emerging from a single clone have been found distributed among numerous Peyer’s patches and intestinal lymph nodes and among upper and lower respiratory tract tissues^48–50^. Our ability to query multiple tissues from the same subject also reveals immunologic connections among the lymphoid tissues of the upper respiratory tract. Additional analyses may help determine whether reseeding of germinal centers and additional rounds of SHM occur in migrating B cell clones^51,52^; recent analyses of B cell lineages following seasonal influenza vaccination provide a framework to test this hypothesis^53^. Further work may also help determine whether persistence of antigen in the tissue correlates with this B cell migration and evolution. The availability of tonsil and adenoid tissues may facilitate such studies.

A limitation of our study is lack of information about the date of infection and presence of symptoms in many participants due to their lack of awareness of having COVID-19. In addition, we do not have longitudinal samples over time from participants to precisely map the duration of immunologic changes; instead, we relied on time from positive PCR/antigen testing to surgery as a proxy. We were also unable to clearly delineate antigen-specific T cells in the tonsils and adenoids likely due to the activated nature of T cells in their chronically inflamed environment. Given the increase in activated populations we observe, it is possible that the expanded Tfh and CD8^+^ T cells we found in the tissue may develop in part as a result of bystander activation during the anti-viral response. Lastly, COVID-19-convalescent participants underwent tonsillectomy for sleep disordered breathing or obstructive sleep apnea due to hypertrophy of the adenoids and/or tonsils, which may be an immunologic disorder^54^. Although control samples also came from individuals with the same conditions, it is possible that these chronic disease states influence the immune response to SARS-CoV-2.

Our findings offer insights into how viral infections may shape the mucosal immune tissue in children beyond the acute phase of infection; maintenance of activated tissue-resident T cells may affect responses against future infectious insults. These cells may also be involved in pathologic responses. It is possible that enrichment of these activated cells in the tissue into convalescence plays a role in delayed or prolonged sequelae of COVID-19 including multisystem inflammatory syndrome in children (MIS-C) or long-haul COVID-19. Children with MIS-C have a high frequency of HLA-DR^+^CD38^+^ CD8^+^ T cells and IFN-γ-induced signatures in the peripheral blood and have mucocutaneous findings including pharyngeal erythema, raising the question of whether type 1 skewed TRM cells may be involved^55–57^. The repository of pharyngeal tissues we have generated may facilitate evaluation of these and other important questions.

## Methods

### Ethics statement

This study was approved by the Institutional Review Board (IRB) at Children’s National Hospital (IRB protocol number 00009806). Written informed consent was obtained from parent/guardians of all enrolled participants, and assent was obtained from minor participants over 7 years of age.

### Participant recruitment

We recruited 110 children who underwent tonsillectomy and/or adenoidectomy at Children’s National Hospital (CNH) in Washington, DC, USA. All children scheduled to undergo tonsillectomy at CNH were eligible. The first 102 participants were recruited from late September 2020 to early February 2021 without screening for prior COVID-19. An additional 2 participants were subsequently recruited with known history of COVID-19, plus 6 additional subjects (one of whom turned out to be positive by serology) were recruited in May and June 2021. Because not all tissues or blood were available from each subject, we collected a total of 106 blood samples, 100 adenoids, and 108 tonsils from 110 participants (Supplementary Table 2). No statistical methods were used to predetermine sample size. All participants had negative RT-PCR testing from a nasopharyngeal swab for SARS-CoV-2 within 72 hours of the surgery. Demographic information and clinical data were collected through parental questionnaires and chart review and inputted and managed in REDCap, and biologic samples were acquired in the operating room by a separate clinical team at CNH.

Eleven participants had previous confirmed SARS-CoV-2 infection with RT-PCR or antigen testing from nasopharyngeal swabs. Another thirteen COVID-19-exposed participants were identified through serum antibody testing and/or identification of B cells that recognize the spike protein of SARS-CoV-2 by flow cytometry (described below). One participant (CNMC 43) had SARS-CoV-2 detected by RT-PCR from the nasopharynx 20 days prior to surgery but had negative serology and no SARS-CoV-2 specific B cells in the tissue or blood. We excluded this subject from our subsequent analysis.

### Control selection within the cohort

Controls for flow cytometric analyses were selected among subjects with no serologic or cellular evidence of prior COVID-19. The primary indication for tonsillectomy in all 24 participants with prior COVID-19 was adenotonsillar hypertrophy leading to sleep disordered breathing (SDB) or obstructive sleep apnea (OSA) (Supplementary Table 1 and 3) except one participant who had eustachian tube dysfunction. Patients with SDB and OSA both have breathing difficulties during sleep (primarily snoring); however, patients with OSA had polysomnography documenting an apnea-hypopnea index greater than 1, while those with SDB did not undergo polysomnography testing and were diagnosed by clinical history alone. None of the 24 participants with COVID-19 had frequent recurrent tonsillitis (more than 6 episodes in a year) or other medical problems that directly affect the immune system aside from atopic disease, nor did they take immunomodulating medications aside from nasal/inhaled steroid or loratadine within 2 weeks of surgery. Therefore, subjects were excluded from the control group if they (a) had periodic fever, recurrent tonsillitis or chronic tonsillitis as primary indication for surgery (N = 15); (b) had more than 6 episodes of tonsillitis in a year (N = 2); (c) took immunomodulatory medications (including montelukast and cetirizine) aside from inhaled steroid or loratadine within 2 weeks of surgery (N = 9); (d) had sickle cell anemia (N = 3), or (e) did not have flow cytometry studies performed on their samples on the day of processing due to sample collection prior to panel finalization or technical problems with the flow cytometer on the day of acquisition. Controls were also excluded if they had indeterminate serologic testing for SARS-CoV-2 infection and did not have any SARS-CoV-2 specific B cells in the tissue or blood (N = 2); both of these participants subsequently had negative neutralizing titers to SARS-CoV-2 as well. Samples included in unsupervised and manual gating analyses of flow cytometry data are listed in Supplementary Table 2.

### Blood and tissue collection

Blood samples were obtained just prior to the surgical procedure in the operating room in serum separator tubes (BD) for serum collection and sodium heparin tubes (BD) for peripheral blood mononuclear cells (PBMCs) extraction from an intravenous line placed for anesthesia. Once received in the laboratory on the day of collection, serum separator tubes were spun at 1200g for 10 min, and serum was aliquoted and stored at −80°C. PBMCs were isolated the day after collection by density gradient centrifugation (Lymphocyte Separation Medium, MP Biomedicals) at 1500rpm for 30 min at room temperature with no brake and washed with PBS. If red blood cell contamination was present, cells were lysed with ACK buffer.

Tonsil and adenoid tissues were stored in RPMI media with 5% FBS (VWR), gentamicin 50mg/mL (Gibco), and 1X antibiotic/antimycotic solution (Gibco) on ice immediately after collection. Tissues were processed the day after collection. A 3–5mm portion of tonsil and adenoid tissue was cut and fixed in 5mL of 10% buffered formalin (Avantik) for 24–48 h. The fixed tissue was then incubated in 70% ethanol until it was paraffin-embedded. The remainder of the tissue was mechanically disrupted and filtered through a 100μm cell strainer to create a single cell suspension, lysed with ACK buffer (Gibco), and washed with PBS three times. Freshly isolated PBMCs and tonsil and adenoid cells were surface stained and analyzed with flow cytometry as described below on the day of processing. The remaining cells were stored in liquid nitrogen in the presence of FBS (VWR) with 10% DMSO.

### SARS-CoV-2 serum antibody ELISA

After thawing frozen serum to room temperature, IgG and IgM antibodies against the spike (S) protein and receptor-binding domain (RBD) of the S protein of SARS-CoV-2 were analyzed using ELISA as previously described^38,58^. Positivity thresholds were based on mean optical density (absorbance) plus 3 standard deviations. The final criterion of S+ and RBD+ for any combination of positive IgG or IgM gave estimated sensitivity and specificity of 100% based on prior studies of this assay. Data are shown in Supplementary Table 4.

### Pseudovirus neutralization assay

Antibody preparations were evaluated by SARS-CoV-2 pseudovirus neutralization assay (PsVNA) using WA-1, B.1.429 (epsilon), B.1.1.7 (alpha), P.1 (gamma), B.1.351 (beta), B.1.526 (iota), B.1.617.2 (delta), and B.1.1.529 (omicron) strains. The PsVNA using the 293-ACE2-TMPRSS2 cell line was described previously^59–61^.

Briefly, human codon-optimized cDNA encoding SARS-CoV-2 S glycoprotein of the WA-1, B.1.429, B.1.1.7, P.1, B.1.351, B.1.526, B.1.617.2, and B.1.1.529 strains were synthesized by GenScript and cloned into eukaryotic cell expression vector pcDNA 3.1 between the BamHI and XhoI sites. Pseudovirions were produced by co-transfection Lenti-X 293T cells with psPAX2(gag/pol), pTrip-luc lentiviral vector and pcDNA 3.1 SARS-CoV-2-spike-deltaC19, using Lipofectamine 3000. The supernatants were harvested at 48h post transfection and filtered through 0.45μm membranes and titrated using 293T-ACE2-TMPRSS2 cells (HEK 293T cells that express ACE2 and TMPRSS2 proteins).

For the neutralization assay, 50 μL of SARS-CoV-2 S pseudovirions were pre-incubated with an equal volume of medium containing serum at varying dilutions at room temperature (RT) for 1 h, then virus-antibody mixtures were added to 293T-ACE2-TMPRSS2 cells in a 96-well plate. The input virus with all SARS-CoV-2 strains used in the current study were the same (2×10^5^ Relative light units/50 μL/well). After a 3 h incubation, the inoculum was replaced with fresh medium. Cells were lysed 24 h later, and luciferase activity was measured using luciferin. Controls included cells only, virus without any antibody and positive sera. The cut-off value or the limit of detection for the neutralization assay is 1:10. Data are shown in Supplementary Table 4.

### High-dimensional flow cytometry

#### SARS-CoV-2 antigen specific B cell detection

5 million cells per sample of PBMC, adenoid, or tonsil were resuspended in PBS with 2% FBS and 2 mM EDTA (FACS buffer). Biotinylated S1 and RBD probes (BioLegend) were crosslinked with fluorochrome-conjugated streptavidin in a molar ratio of 4:1. Fluorochrome-conjugated streptavidin was split into 5 aliquots and conjugated to biotinylated S1 and RBD probes by mixing for 20 min/aliquot at 4°C. Cells were first stained with the viability dye, Zombie NIR (1:800 dilution, BioLegend), for 15 min at RT, washed twice and then incubated with True-Stain Monocyte Blocker (BioLegend) for 5 min. An antibody cocktail containing the rest of the surface antibodies, the fluorochrome-conjugated S1 and RBD probes, and Brilliant Stain Buffer Plus (BD) were then added directly to the cells and incubated for 30 min at RT in the dark (200uL staining volume). Cells were washed three times and fixed in 1% paraformaldehyde for 20 min at RT before washing again and collecting on a spectral flow cytometer (Aurora, Cytek). Antibodies used in this assay are shown in Supplementary Table 8.

#### Broad 37 parameter immunophenotyping flow cytometry panel

2 million cells per sample of PBMC and 5 million cells per adenoid or tonsil were resuspended in FACS buffer. Cells were first stained with LIVE/DEAD Blue (1:800, ThermoFisher) for 15 min at RT, washed twice and then incubated with True-Stain Monocyte Blocker (BioLegend) for 5 min. Antibodies for chemokine receptors and TCRγδ were sequentially added at RT (anti-CCR7 for 10 min, anti-CCR6, anti-CXCR5 and anti-CXCR3 together with Brilliant Stain Buffer Plus for 5 min, anti-TCRγδ for 10 min). An antibody cocktail containing the rest of the surface antibodies and Brilliant Stain Buffer Plus were then added directly to the cells and incubated for 30 min at RT in the dark (total staining volume 182uL). Cells were washed three times and stained with fluorescence conjugated streptavidin for 15 min at RT. Then, cells were washed twice and fixed in 1% paraformaldehyde for 20 min at RT before washing again and acquiring on the Aurora spectral cytometer (Cytek). Antibodies used in this assay are shown in Supplementary Table 8. The frequency of major populations was determined using FlowJo Software v10 (BD Biosciences) based on previously described manual gating strategies^62^.

#### Unsupervised analysis of flow cytometry data and statistical modelling of meta-clustering results

Data from the broad immunophenotyping flow cytometry panel with 37 parameters were analyzed with unsupervised clustering of surface antibody staining. CD19^+^ B cells, CD4^+^ T cells, and CD8^+^ T cells were analyzed separately. Tonsil and adenoid samples were merged and processed together while PBMC samples were processed separately due to pre-determined antibody concentration differences in staining required for optimal results in each organ. B cell analysis was based on surface expression of CCR6, CXCR5, CXCR3, CCR7, CD45RA, CD11c, IgD, CD20, IgM, IgG, CD27, HLA-DR, CD38, CD21, CD123, PD-1, CD57, CD25, CD24, CD95, IgA, CD1c, CD127 and CD161. CD4^+^ and CD8^+^ T cells analyses were based on the expression of CCR6, CXCR5, CXCR3, CCR7, CD45RA, CD161, CD28, PD-1, CD57, CD25, CD95, CD27, CD127, HLA-DR, CD38, ICOS, CD11c, CD24, CD1c, CD123, and CD21. FCS files (3.0) as well as FlowJo workspaces (10.7.2) were processed in R (4.1) via Rstudio (1.4.1717) and Bioconductor (3.13) using cytoverse (0.0.0.9000), including flowCore (2.4.0), flowWorkspace (4.4.0), ggcyto (1.20.0), openCyto (2.4.0), CytoML (2.4.0), cytolib (2.4.0) and cytoqc (0.99.2). Default options for biexponential data transformation were used. Outlier cells with expression values in the top or bottom 1e-3 quantiles were excluded. Single cells in each sample were first clustered using k-means (k = 500, referred to as metacells), followed by merging cluster centroids from different samples with the same staining (i.e., tonsil/adenoids vs PBMC) for meta clustering and dimensionality reduction. Specifically, 500 centroids from each sample (metacells) were merged followed by another run of k-means meta-clustering (again k = 500), which were finally used in Leiden clustering and to learn a t-UMAP model to project the metacells (i.e., single cell-level k-means centroids; shown in plots). Seurat (4.0.3), uwot (0.1.10), and leiden (0.3.9) were used in shared nearest neighbors graph building, t-UMAP projection, and meta-clustering, respectively, with default settings. Leiden meta-clusters were mapped back to the single cell level and the ranked frequency of single cells in each Leiden meta-cluster in each sample was modeled linearly as a function of age, sex, and history of COVID-19 (COVID status) (as in lm(rank(frequency) ~ age + sex + status). Prior to statistical modeling, PCA of frequencies was used to detect and exclude outlier samples. Sample sizes are described in the legend of each plot. t-UMAP projections as well as all confidence intervals of coefficients and their p-values (from two-tailed t-test of each coefficient within each model) are presented in plots built with ggplot2 (3.3.5). Data are shown in Supplementary Table 10.

### Single cell RNA sequencing

#### Processing for CITE-seq

Banked PBMC, tonsils and adenoids from 2 donors with history of COVID-19 (CNMC 71 and 89) and one control (CNMC 99) were thawed from liquid nitrogen in a 37°C water bath for 2–3 mins. 2 mL of media consisting of RPMI with 10% of fetal bovine serum, 0.1mg/ml DNase I (Roche) and 10mM HEPES was added drop-by-drop to the thawed cells. Cells were further diluted by incremental addition of a 1:1 volume of media up to 8 mL, then centrifuged at 1600 rpm for 5 min. Cells were then resuspended in 300 μL of media, incubated at RT for 5 min, washed with media without DNase I, and filtered through a 100μm strainer before spinning down for culture and resuspending in staining buffer (PBS + 1% BSA). Cells were then incubated with Fc blocker (Human TruStain FcX, BioLegend), stained with TotalSeq-C human hashtag antibodies (BioLegend) to uniquely label the sample origin (by tissue and donor), and washed with PBS + 0.04% BSA. Adenoids and tonsils from the 3 donors (6 samples in total) were pooled together and PBMCs from 3 were pooled together separately. The number of cells to pool from each tissue and donor was calculated with the aim of pooling a similar number of S1^+^ positive B cells from each sample. Pooled cells were first incubated with Fc blocker at 4°C for 10 min followed by CITE-seq and sorting antibody cocktails in the following order at 4°C: TotalSeq anti-CXCR3 antibody for 10 min, TotalSeq chemokine cocktail (anti-CCR7, CCR6, CXCR5 antibodies) for 10 min, and the rest of CITE-seq antibodies and fluorescence-labeled sorting antibodies and viability dye (Aqua) for 30 min (Supplementary Table 8). Cells were then washed with PBS+0.04% BSA and resuspended in PBS+2% FBS. S1^+^ and S1^−^ B cells were sorted from each pool on a BD FACS Aria Fusion sorter for tonsil/adenoid pool and FACS Aria Illu sorter for the PBMC pool (BD Biosciences, San Jose, CA). See Supplementary Figure 3 for sorting strategy. Cells were sorted into PBS+2% FBS. Note that the antibody concentrations used for CITE-seq were optimized by the manufacturer based on healthy PBMC samples, and thus may not be optimal for tissue samples. We have not independently verified the specificity of each antibody in our CITE-seq panel. Antibody concentrations were based on our titration from flow cytometry^63,64^.

Sorted S1^+^ and S1^−^ B cells were mixed with the reverse transcription mix and partitioned into single cell Gel-Bead in Emulsion (GEM) using 10× 5’ Chromium Single Cell Immune Profiling Next GEM v2 chemistry (10× Genomics, Pleasanton, CA). The reverse transcription step was performed in an Applied Materials Veriti 96-well thermocycler. 10× Genomics 5’ single cell gene expression, cell surface protein, and B cell receptor (BCR) libraries were prepared as instructed by 10× Genomics user guides (https://www.10xgenomics.com/resources/user-guides/). RNA quality and quantity in the libraries were measured using a bioanalyzer (Agilent, Santa Clara, CA) and a Qubit fluorometer (ThermoFisher). Libraires were pooled at a concentration of 10nM and sequenced on Illumina NovaSeq platform (Illumina, San Diego, CA) using the following read lengths: Read 1: 26 base pairs, Index 1: 10 base pairs, Index 2: 10 base pairs, Read 2: 150 base pairs.

#### CITE-seq data processing and analysis

CellRanger (10x Genomics) version 6.0.0 was used to map cDNA libraries to the hg19 genome reference (10x genomics hg19 cellranger reference, version 1.2.0) and to count antibody tag features. Data were further processed using Seurat (v.4.0.1)^65^ running in R v4.0.3. After transforming the surface protein library counts using *dsb*^66^, we demultiplexed the pooled samples using manual cutoffs on the hashtag antibody staining. We removed cells with less than 100 detected genes, greater than 30% mitochondrial reads, or mRNA counts greater than 25,000. To exclude cells with extremely high surface antibody counts, we also removed the top 0.05% of cells in the surface antibody total count distribution. Cell clustering was performed by applying the FindNeighbors() function from *Seurat* on a distance matrix generated from the *dsb*-transformed surface protein data, followed by Louvain clustering on the resulting SNN graph using *Seurat’s* FindClusters() algorithm, with a resolution parameter of 1. Expression of selected genes were visualized using the *ComplexHeatmap* package^67^, and the percentage of cells per cluster for the S1^+^ and S1^−^ cells was plotted using *ggplot2*^68^. For the comparison of differentially expressed genes between the S1^+^ and S1^−^ B cells, we first downsampled the fastq files from the S1^+^ sequencing library to more closely match the reads-per-cell obtained in the S1^−^ sequencing libraries using *seqtk* v1.3. Differential expression was then compared using the MAST algorithm with “Donor” as a latent variable, as implemented in the Seurat *FindMarkers* function. For RNA-based clustering S1^+^ and S1^−^ B cells, we first downsampled the fastq files from the S1^+^ sequencing library to more closely match the reads-per-cell obtained in the S1^−^ sequencing libraries using *seqtk* v1.3. Cells were then clustered using the top 15 PCs derived from the 2000 most variable genes, selected by Seurat’s *FindVariableFeatures* function using the “vst” method. Clustering was performed using the Louvain method and a resolution of 1.15 in Seurat’s *FindClusters* function.

#### BCR sequence analysis and clonal clustering

BCR repertoire sequence data were analyzed using the Immcantation (www.immcantation.org) framework. Starting with filtered CellRanger output, V(D)J genes for each sequence were aligned to the IMGT GENE-DB reference database v3.1.29^69^ using IgBlast v1.16.0^70^ and Change-O v1.0.0^71^. Nonproductive sequences, cells without associated constant region calls, cells identified as arising from doublets or negative wells, and cells with multiple heavy chains were all removed. Samples within each subject were pooled and sequences were grouped into clonal clusters, which contain B cells that relate to each other by somatic hypermutations from a common V(D)J ancestor. Sequences were first grouped by common IGHV gene annotations, IGHJ gene annotations, and junction lengths. Using the hierarchicalClones function of *scoper* v1.1.0^72^, sequences within these groups differing by a length normalized Hamming distance of 0.1 within the CDR3 region were defined as clones using single-linkage hierarchical clustering^73^. This threshold was determined through manual inspection of distance to nearest neighbor plots using *shazam* v1.1.0^74^. These heavy chain defined clonal clusters were further split if their constituent cells contained light chains that differed by V and J genes. Within each clone, germline sequences were reconstructed with D segment and N/P regions masked (replaced with “N” nucleotides) using the createGermlines function within *dowser* v0.1.0^75^. All BCR analyses used R v4.1.1 (R Core Team 2017), and plots were generated using *ggpubr* v0.4.0^76^ and *ggplot2* v3.3.5^68^. After clonal clustering, only heavy chain sequences were used for subsequent analysis. Somatic hypermutation was calculated as the Hamming distance between each sequence’s IMGT-gapped sequence alignment and its predicted unmutated germline ancestor along the V-gene (IMGT positions 1–312).

Clonal diversity is an important metric of B cell repertoires, and low B cell clonal diversity is consistent with an adaptive immune response. To quantify B cell clonal diversity, we calculated Simpson’s diversity for each sample using the alphaDiversity function of *alakazam* v1.1.0^71^. Lower values of Simpson’s diversity indicate a greater probability of two random sequences belonging to the same clone, consistent with more large clones. To account for differences in sequence depth, samples within each comparison were downsampled to the same number of sequences, and the mean of 1000 such re-sampling repetitions was reported. Only donor/tissue/cell sort samples with at least 100 B cells were included, which led to the exclusion of all S1^+^ cells from CNMC 99 (control with no history of COVID-19) and S1^+^ PBMCs from CNMC 89 (COVID-19 convalescent). Clonal overlap among tissues can be used as a measure of immunological connectivity. Clonal overlap was calculated using the Jaccard index, which for each pair of tissues is the number of unique clones found in both tissues (intersect) divided by the total number of unique clones among the two tissues (union). Clones were labelled as “S1^+^” if they contained at least one S1^+^ sorted B cell. To infer lineage trees, we estimated tree topologies, branch lengths, and subject-wide substitution model parameters using maximum likelihood under the GY94 model^77,78^. Using fixed tree topologies estimated from the GY94 model, we then estimated branch lengths and donor-wide parameter values under the HLP19 model in IgPhyML v1.1.3^77^. Trees were visualized using *dowser* v0.1.0^75^ and *ggtree* v3.0.4^79^.

### Whole slide multiplexed imaging of FFPE tissue sections

#### Tissue and slide processing and staining

5 μm tissue sections were cut from FFPE samples and placed onto glass slides. Following sectioning, glass slides (with tissue) were baked in a 60°C oven for 1 hour. Deparaffinization was performed as described previously^80^: 2 exchanges of 100% xylene (10 minutes per exchange) followed by 100% ethanol for 10 minutes, 95% ethanol for 10 minutes, 70% ethanol for 5 minutes, and 10% formalin for 15 minutes. Antigen retrieval was performed by incubating slides in AR6 buffer (Akoya Biosciences) for 40 minutes in a 95°C water bath. After 40 minutes, slides were removed from the water bath and allowed to cool on the bench for 20 minutes. Sections were permeabilized, blocked, and stained in PBS containing 0.3% Triton X-100 (Sigma-Aldrich), 1% bovine serum albumin (Sigma-Aldrich), and 1% human Fc block (BD Biosciences). Immunolabeling was performed with the PELCO BioWave Pro 36500–230 microwave equipped with a PELCO SteadyTemp Pro 50062 Thermoelectric Recirculating Chiller (Ted Pella) using a 2–1-2–1-2–1-2–1-2 program^80,81^. A complete list of antibodies and imaging panels with labelling steps can be found in Supplementary Table 8. In general, primary antibodies were applied first, washed 3 times in PBS, and incubated with appropriate secondary antibodies. Directly conjugated primary antibodies were applied last after blocking with host sera (5%). Endogenous biotin was blocked using the Avidin/Biotin Blocking Kit (Abcam). Cell nuclei were visualized with Hoechst (Biotium) and sections were mounted using Fluoromount G (Southern Biotech).

#### Confocal microscopy, image analysis, and histo-cytometry

Images were acquired using an inverted Leica TCS SP8 X confocal microscope equipped with a 40X objective (NA 1.3), 4 HyD and 1 PMT detectors, a white light laser that produces a continuous spectral output between 470 and 670 nm as well as 405, 685, and 730 nm lasers. All images were captured at an 8-bit depth, with a line average of 3, and 1024×1024 format with the following pixel dimensions: x (0.284 μm), y (0.284 μm), and z (1 μm). Images from whole tissue sections were tiled and merged using the LAS X Navigator software (LAS X 3.5.5.19976). Fluorophore emission was collected on separate detectors with sequential laser excitation of compatible fluorophores (3–4 per sequential) used to minimize spectral spillover. The Channel Dye Separation module within the LAS X 3.5.5.19976 (Leica) was then used to correct for any residual spillover. Threshold identification, voxel gating, surface creation, and masking were performed as previously described using Imaris software (Imaris version 9.8.0, Bitplane AG)^82,83^. For publication quality images, gaussian filters, brightness/contrast adjustments, and channel masks were applied uniformly to all images.

A combination of automatic and manual surface/contour creation methods were used to define germinal center (GC) regions of interest (ROI) with Imaris software (Imaris version 9.8.0, Bitplane AG). GCs were identified as aggregations of 5 or more Ki-67^+^ nuclei. For each sample, whole tissue ROIs were generated using the Hoechst channel and surface function of Imaris. The resulting metric, total area of tissue imaged, was then used to normalize the number and size of GCs between samples.

The number and phenotype of T cells inside and outside of the B cell follicle/GC were quantified using histo-cytometry^83^. Cells were segmented on Hoechst^+^ nuclei and used to create surfaces. Channel statistics for all surfaces were exported into Excel (Microsoft) and converted to a csv file for direct visualization in FlowJo v10.6.1 (Treestar). Mean voxel intensities for all channels were plotted on a linear scale and used for gating distinct lymphocyte populations. B cell follicle and GC gates were defined using positional data on the HLA-DR^+^CD3^−^ and Ki-67^+^ surfaces, respectively. These positional gates were applied to T cell surfaces to calculate the frequency of T cell phenotypes inside or outside of the follicle/GC as demonstrated previously^84,85^. T cell numbers were normalized across all samples to account for differences in the number of T cells analyzed per sample. Imaging data were exported and processed in Excel (Microsoft Office) and GraphPad Prism 8.2.1.

### Activated induced marker (AIM) assay

Banked frozen PBMC and tonsil and adenoid cells were thawed as described above in “Processing for CITE-seq.” Two million mononuclear cells from tonsil or adenoid or one million PBMC from each donor were cultured in a 96 well round bottom plate at a concentration of 1×10^7^ cells/mL in media consisting of RPMI plus 5% human AB serum (Omega), 2 mM L-glutamine, 0.055 mM beta-mercaptoethanol, 1% penicillin/streptomycin, 1 mM sodium pyruvate, 10 mM HEPES, and 1% non-essential amino acids. Cells were blocked at 37°C for 15 min prior to peptide pool stimulation with 0.5μg/mL of anti-CD40 mAb (Miltenyi). Following this, cells were stimulated with SARS-CoV-2 peptide pools for 18 hours at 37°C in 5% CO_2_ incubator. The following peptide pools were reconstituted per instructions and used for stimulation (Miltenyi): PepTivator SARS-CoV-2 Prot_S+, PepTivator SARS-CoV-2 Prot_S1, PepTivator SARS-CoV-2 Prot_S, PepTivator SARS-CoV-2 Prot_N, PepTivator SARS-CoV-2 Prot_M. Prot_S+, Prot_S1 and Prot_S were pooled into one megapool of spike peptides at concentration of 0.6 nmol/ml for each pool. PHA-L (Millipore) at 5μg/ml was used as positive control. Negative control wells lacking peptides were supplemented with an equivalent volume of DMSO and ddH_2_O. After stimulation, cells were first stained with a viability dye (LIVE/DEAD Blue, ThermoFisher) for 15 min at RT, washed twice and then incubated with True-Stain Monocyte Blocker (BioLegend) for 5 min. Antibodies for chemokine receptors (anti-CXCR3 for 10 min, anti-CCR7 for 10 min, anti-CXCR5 and anti-CCR6 together for 5 min) were sequential added at RT. The antibody cocktail containing the rest of the surface antibodies and Brilliant Stain Buffer Plus (BD) was then added directly to the cells and incubated for 30 min at RT in the dark (total staining volume 180uL). Stained cells were washed three times and fixed in 1% paraformaldehyde for 20 min at RT before collecting on the Aurora spectral cytometer (Cytek). Antibodies and reagents used in this assay are listed in Supplementary Table 8.

### T cell functional assays - intracellular cytokine staining

Frozen cells were thawed as described in “Processing for CITE-seq.” 2 million PBMC, adenoid, or tonsil cells from each sample were resuspended in 200 μL of complete RPMI medium containing 10% FBS (VWR), 2 mM glutamine, 0.055 mM beta-mercaptoethanol, 1% penicillin/streptomycin, 1 mM sodium pyruvate, 10 mM HEPES, and 1% non-essential amino acids. Cells were stimulated with PMA (50ng/ml, Sigma) and ionomycin (1000ng/ml, Sigma) for 2.5 h in the presence of anti-CD107a (BioLegend), GolgiSTOP (monensin, BD), and GolgiPlug (BFA, BD). After stimulation, surface markers were stained as described above in the AIM assay. Surface-stained cells were washed and fixed with Cytofix Fixation Buffer (BD) at RT for 20 min and washed with permeabilization buffer (eBioscience) twice. Then, the intracellular cytokine antibody mix was added for 30 min at RT (staining volume 50uL). Stained cells were collected on the Aurora spectral cytometer (Cytek). Antibodies used in this assay are listed in Supplementary Table 8.

### Viral quantification in FFPE blocks by ddPCR

RNA was extracted from scrolls cut from FFPE tonsil and adenoid tissues using the RNeasy FFPE Kit (Qiagen) according to the manufacturer’s protocol. A NanoDrop ND-1000 Spectrophotometer (Thermo Fisher Scientific) was used to quantify RNA concentrations. The QX200 AutoDG Droplet Digital PCR System (Bio-Rad) was used to detect and quantify SARS-CoV-2 RNA using the SARS-CoV-2 Droplet Digital PCR Kit (Bio-Rad), which contains a triplex assay of primers/probes aligned to the CDC markers for SARS-CoV-2 N1 and N2 genes and human *RPP30* gene. Ninety-six-well plates were prepared with technical replicates of up to 550 ng of RNA per well using the aforementioned kit according to the manufacturer’s instructions. The QX200 Automated Droplet Generator (Bio-Rad) provided microdroplet generation, and plates were sealed with the PX1 PCR Plate Sealer (Bio-Rad) before proceeding with RT–PCR on the C1000 Touch Thermal Cycler (Bio-Rad) according to the manufacturer’s instructions. Plates were read on the QX200 Droplet Reader (Bio-Rad) and analyzed using the freely available QuantaSoft Analysis Pro Software (Bio-Rad) to quantify copies of N1, N2 and RP genes per well, which was then normalized to RNA concentration input. For samples to be considered positive for SARS-CoV-2 N1 or N2 genes, they needed to average the manufacturer’s limit of detection of ≥ 0.1 copies per μl and two positive droplets per well.

### Statistics and reproducibility

Please see above for a detailed description of statistical analysis of results from unsupervised analysis as well as where to find reproducible scripts. Simplified Presentation of Incredibly Complex Evaluation (SPICE) software (version 6, NIAID, NIH, Bethesda, MD, USA, https://niaid.github.io/spice/) was used to analyze flow cytometry data on T cell polyfunctionality^30^. Graphs were produced by Prism (v8). Statistical analyses were performed using SPSS (IBM, version 28.0.0.0). Differences between groups were compared using the Mann-Whitney U test for independent values and Wilcoxon signed ranks test for paired values. Correlations were assessed using the Spearman rank correlation. All statistical tests were two-sided. p<0.05 was considered significant.

## Extended Data

**Extended Figure Data 1.**
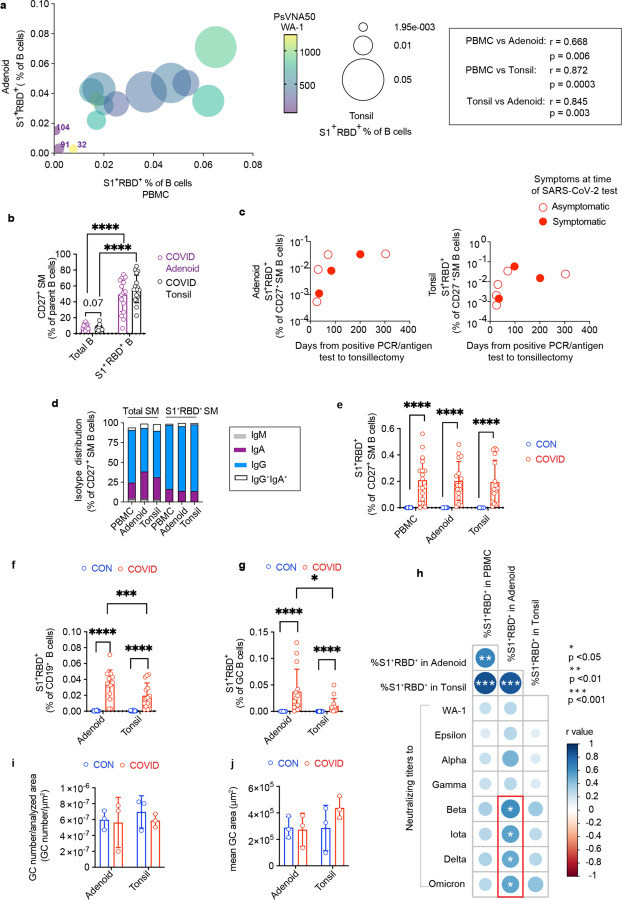
Characterization of neutralization titers and S1^+^RBD^+^ B cells a. Correlation among frequencies of S1^+^RBD^+^ cell among B cells in peripheral blood, tonsils, and adenoids. The color of data points indicates neutralizing titers (PsVNA50) to the WA-1 variant. Donors 32, 91, 104, who had the lowest frequencies of S1^+^RBD^+^ B cells, are labeled in the plot. b. Frequency of CD27^+^ switched memory (SM) B cells among total B cells and among S1^+^RBD^+^ B cells from adenoid and tonsil samples from COVID-19-convalescent participants c. Frequency of S1^+^RBD^+^ cells among CD27^+^ SM B cells in adenoid and tonsil according to time from presumed active infection (positive PCR/antigen test from nasopharyngeal swab) to surgery d. Proportion of each isotype among S1^+^RBD^+^ SM B cells and total SM B cells in PBMC, adenoid, and tonsil following COVID-19. The percentage of IgA^+^ cells was significantly lower among S1^+^RBD^+^ SM B cells compared to total SM B cells in the tissue (p < 0.0001 for adenoid, p < 0.0001 for tonsil). e. Percentage of S1^+^RBD^+^ cells among CD27^+^ SM B cells from PBMC, adenoid, and tonsil following COVID-19 (COVID) vs. controls (CON) f and g. Percentage of S1^+^RBD^+^ cells among total B cells (f) and GC B cells (g) from 14 pairs of adenoid and tonsil COVID samples vs. CON h. Summary of correlations among neutralizing titers (PsVNA50) against several SARS-CoV-2 variants and frequencies of S1^+^RBD^+^ cells among B cells in peripheral blood, adenoids, and tonsils. i. and j. Mean number of GCs per total scanned tissue area (i) and mean GC area (total GC area in section/total number of GCs in section) (j) from adenoid and tonsil in COVID vs. CON samples. Samples imaged are in Supplementary Table 9. Gating strategy is shown in Supplementary Fig. 1–2. Samples used in panels a-h are listed in Supplementary Table 2 and 4 (PBMC COVID n = 18, CON n = 33; adenoid COVID n = 16, CON n = 27; and tonsil COVID n = 16, CON n = 30). Each symbol represents data from one donor. Means ± S.D. are displayed in the scatter and bar plots. Significance calculated with Mann-Whitney U test for unpaired values or Wilcoxon signed ranks test for paired values from the same donor. Correlation analysis performed with Spearman’s rank correlation. * p<0.05, *** p<0.001, **** p<0.0001.

## Supplementary Material

Supplement 1

Supplement 2

Supplement 3

Supplement 4

Supplement 5

Supplement 6

Supplement 7

Supplement 8

Supplement 9

Supplement 10

Supplement 11

Supplement 12

Supplement 13

Supplement 14

Supplement 15

Supplement 16

Supplement 17

Supplement 18

Supplement 19

Supplement 20

Supplement 21

Supplement 22

## Data Availability

As sequencing data were collected from children, who are considered a vulnerable population, raw CITE-seq data are available upon request to corresponding authors. All other data are provided with the article or upon request from the corresponding authors. Source data for figures will be provided with this article.
