# Supplementary material for: Robust, persistent adaptive immune responses to SARS-CoV-2 in the oropharyngeal lymphoid tissue of children
Source: Res Sq. 2022 Mar 23:rs.3.rs-1276578. Preprint. [Version 1] doi: 10.21203/rs.3.rs-1276578/v1 (PMC8963700; doi:10.21203/rs.3.rs-1276578/v1)
Supplement: Supplement 4 [file 127deb7093e8518a6ea3cfd2.docx]

**Supplemental Table 1: Demographic characteristics of participants**

**All Participants COVID-19 + COVID-19 -**

n=110 n=24 n=86

**Demographics**

**Mean Age (years) (±SD)** 6.84 ($\pm4.4$) 6.22 ($\pm$3.2) 7.01 ($\pm$4.7)

**Sex**

Male 61 (55.5%) 13 (54.2%) 48 (55.8%)

Female 49 (44.5%) 11 (45.8%) 38 (44.2%)

**Race/Ethnicity**

Black 27 (24.5%) 3 (12.5%) 24 (27.9%)

Hispanic 42 (38.2%) 18 (75.0%) 24 (27.9%)

White 26 (23.6%) 0 (0.0%) 26 (30.2%)

Other/Mixed 15 (13.6%) 3 (12.5%) 12 (14.0%)

**Clinical Characteristics**

**Primary Diagnosis**

Sleep disordered breathing 60 (54.5%) 14 (58.3%) 46 (53.5%)

Mild OSA, AHI >1 and < 5 14 (12.7%) 6 (25.0%) 8 (9.3%)

Moderate OSA, AHI 5 to 10 6 (5.5%) 1 (4.2%) 5 (5.8%)

Severe OSA, AHI >10 14 (12.7%) 2 (8.3%) 12 (14.0%)

Eustachian tube dysfunction 1 (0.9%) 1 (4.2%) 0 (0.0%)

PFAPA 7 (6.4%) 0 (0.0%) 7 (8.1%)

Recurrent tonsillitis 3 (2.7%) 0 (0.0%) 3 (3.5%)

Chronic tonsillitis/tonsil stones 5 (4.5%) 0 (0.0%) 5 (5.8%)

**Medications (within 2 weeks prior to surgery)**

Inhaled or nasal corticosteroid 14 (12.7%) 4 (16.7%) 10 (11.6%)

Oral corticosteroid 7 (6.4%) 0 (0.0%) 7 (8.1%)

Loratadine (Claritin) 10 (9.1%) 1 (4.2%) 9 (10.5%)

Montelukast (Singulair) 6 (5.5%) 0 (0.0%) 6 (7.0%)

Cetirizine (Zyrtec) 5 (4.5%) 0 (0.0%) 5 (5.8%)

**Prior COVID-19 infection by serology/flow cytometry**

Negative 86 (78.2%) 0 (0.0%) 86 (100%)

Positive 24 (21.8%) 24 (100%) 0 (0.0%)

OSA = Obstructive sleep apnea

AHI = Apnea hypopnea index obtained by polysomnography

PFAPA = periodic fever, aphthous stomatitis, pharyngitis, adenitis syndrome
