# Supplementary material for: Robust, persistent adaptive immune responses to SARS-CoV-2 in the oropharyngeal lymphoid tissue of children
Source: Res Sq. 2022 Mar 23:rs.3.rs-1276578. Preprint. [Version 1] doi: 10.21203/rs.3.rs-1276578/v1 (PMC8963700; doi:10.21203/rs.3.rs-1276578/v1)
Supplement: Supplement 7 [file 41df0ecf827bf300c706c615.docx]

| **Patient ID** | **Age (years)** | **Sex** | **Reason for tonsillectomy/**  **adenoidectomy** | **Medication** | **Co-morbid conditions** | **­­­­­Prior positive SARS-CoV-2 PCR/Ag test** | **Days from positive PCR/Ag to surgery** | **Symptomatic at time of positive PCR/Ag test?** | **Symptoms at time of positive PCR/Ag test** |
| --- | --- | --- | --- | --- | --- | --- | --- | --- | --- |
| CNMC 001 | 2.6 | F | SDB |  |  | No |  |  |  |
| CNMC 005 | 7.5 | F | SDB |  | Asthma | No |  |  |  |
| CNMC 008 | 3.4 | M | Mild OSA |  |  | No |  |  |  |
| CNMC 011 | 8 | M | Mild OSA |  | Asthma | Yes | 142 | Symptomatic | Headache, sore throat, diarrhea, myalgia |
| CNMC 016 | 8.9 | F | SDB |  |  | No |  |  |  |
| CNMC 022 | 4.1 | M | Mild OSA |  |  | Yes | 108 | Symptomatic | Cough, myalgia |
| CNMC 029 | 3.1 | M | SDB |  |  | No |  |  |  |
| CNMC 032 | 3.6 | F | Severe OSA |  |  | Yes | 26 | Asymptomatic |  |
| CNMC 041 | 6 | F | Severe OSA |  |  | No |  |  |  |
| CNMC 046 | 7.2 | M | SDB |  |  | No |  |  |  |
| CNMC 050 | 2.9 | F | SDB | Inh Steroid |  | Yes | 31 | Asymptomatic |  |
| CNMC 069 | 3.4 | F | SDB | Inh Steroid |  | Yes | 201 | Symptomatic | Shortness of breath, myalgia, dizziness, GI symptoms |
| CNMC 070 | 16.4 | M | Mild OSA |  |  | Yes | 98 | Symptomatic | Fever, cough, anosmia, myalgia, GI symptoms, chest pain |
| CNMC 071 | 6.2 | F | SDB |  |  | Yes | 71 | Symptomatic | Fever, cough, shortness of breath, anosmia, myalgia, GI symptoms |
| CNMC 087 | 6 | M | SDB | Loratadine |  | No |  |  |  |
| CNMC 089 | 12.1 | F | SDB |  |  | No |  |  |  |
| CNMC 091 | 9.2 | M | Moderate OSA |  |  | No |  |  |  |
| CNMC 100 | 4.7 | F | Mild OSA | Inh Steroid |  | Yes | 25 | Asymptomatic |  |
| CNMC 101 | 4.6 | M | SDB |  |  | No |  |  |  |
| CNMC 102 | 5.1 | M | SDB |  |  | Yes | 35 | Symptomatic | Fever, myalgia |
| CNMC 103 | 8.1 | M | Mild OSA |  |  | Yes | 303 | Asymptomatic |  |
| CNMC 104 | 6.8 | M | SDB | Inh Steroid |  | No |  |  |  |
| CNMC 108 | 4.2 | F | ETD |  |  | Yes | 84 | Symptomatic | Cough, diarrhea, nasal congestion |
| CNMC 109 | 5.1 | M | SDB |  |  | No |  |  |  |

**Supplementary Table 3: Characteristics of Participants with Prior COVID-19**

SDB: sleep disordered breathing

OSA: obstructive sleep apnea

ETD: eustachian tube dysfunction

Ag: antigen Inh Steroid: inhaled steroid GI: gastrointestinal

F: female M: male
