# Supplementary material for: Robust, persistent adaptive immune responses to SARS-CoV-2 in the oropharyngeal lymphoid tissue of children
Source: Res Sq. 2022 Mar 23:rs.3.rs-1276578. Preprint. [Version 1] doi: 10.21203/rs.3.rs-1276578/v1 (PMC8963700; doi:10.21203/rs.3.rs-1276578/v1)
Supplement: Supplement 18 [file deead97d62ce47c0e6cf8df3.pdf]

Supplementary Figure 1

Gating strategy for Tonsil and Adenoid

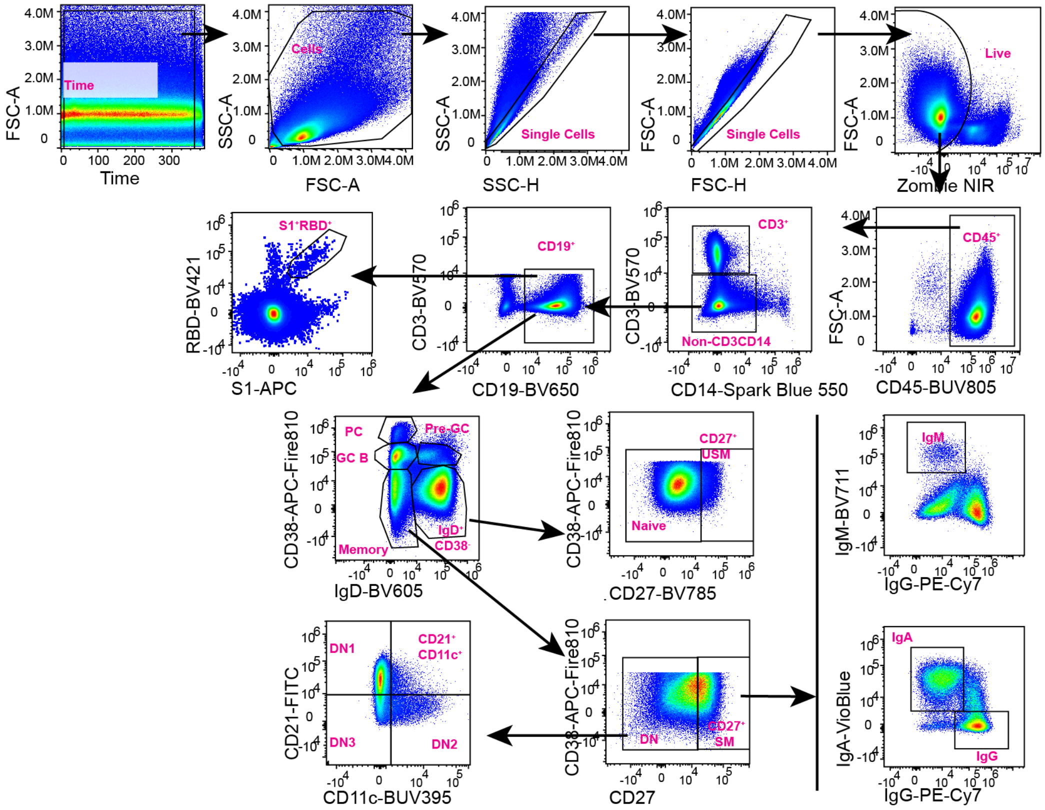

Supplementary Figure 1. Gating strategy of major CD19⁺ B cell populations and S1⁺RBD⁺ B cells in pharyngeal tissues

Representative flow cytometry plots of major B cell populations and S1⁺RBD⁺ B cell gating strategy in pharyngeal tissues, including PC (plasma cells), GC (germinal center B cells), pre-GC (pre-germinal center B cells), naïve (naïve B cells), DN (double negative B cells), CD27⁺ SM (CD27⁺ switched memory B cells), CD27⁺ USM (CD27⁺ unswitched memory cells). Gating for DN subsets is also shown.

Supplementary Figure 2

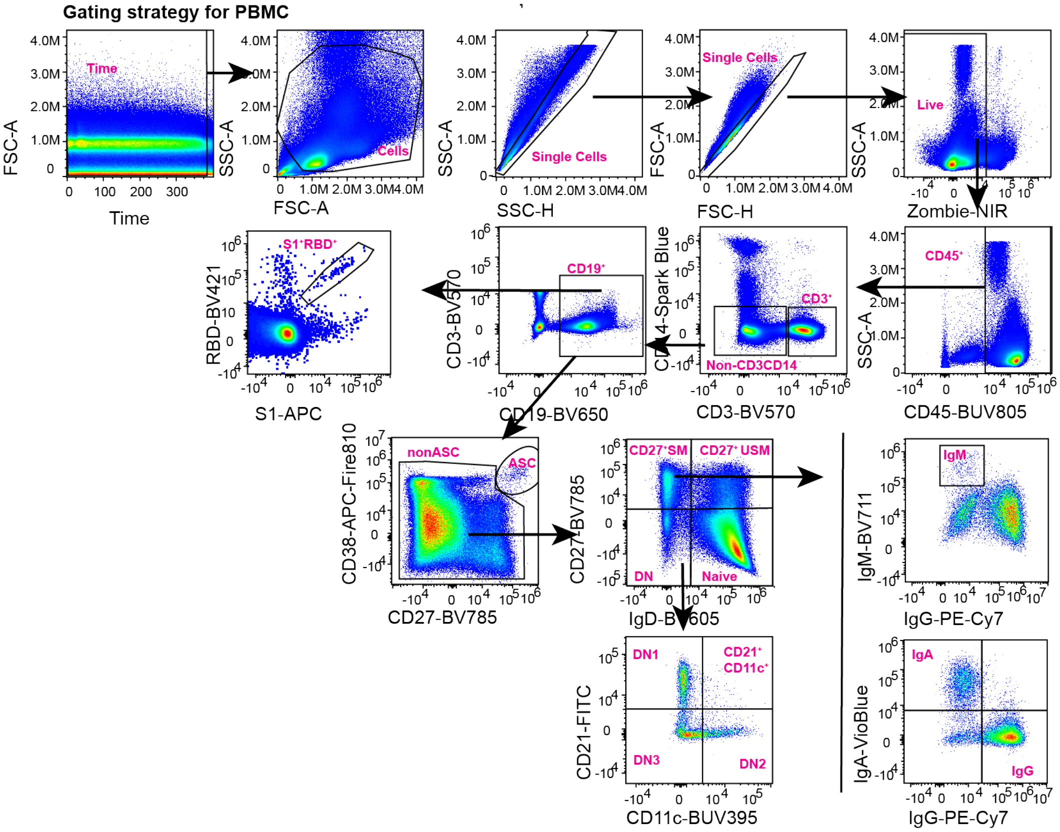

Supplementary Figure 2. Gating strategy of major CD19⁺ B cell populations and S1⁺RBD⁺ B cells in peripheral blood

Representative flow cytometry plots of major B cell populations and S1⁺RBD⁺ B cell gating strategy in peripheral blood, including ASC (antibody secreting cells equivalent to plasma cells and plasmablasts), naïve (naïve B cells), DN (double negative B cells), CD27⁺ SM (CD27⁺ switched memory B cells), CD27⁺ USM (CD27⁺ unswitched memory B cells). Gating for DN subsets is also shown.

Supplementary Figure 3

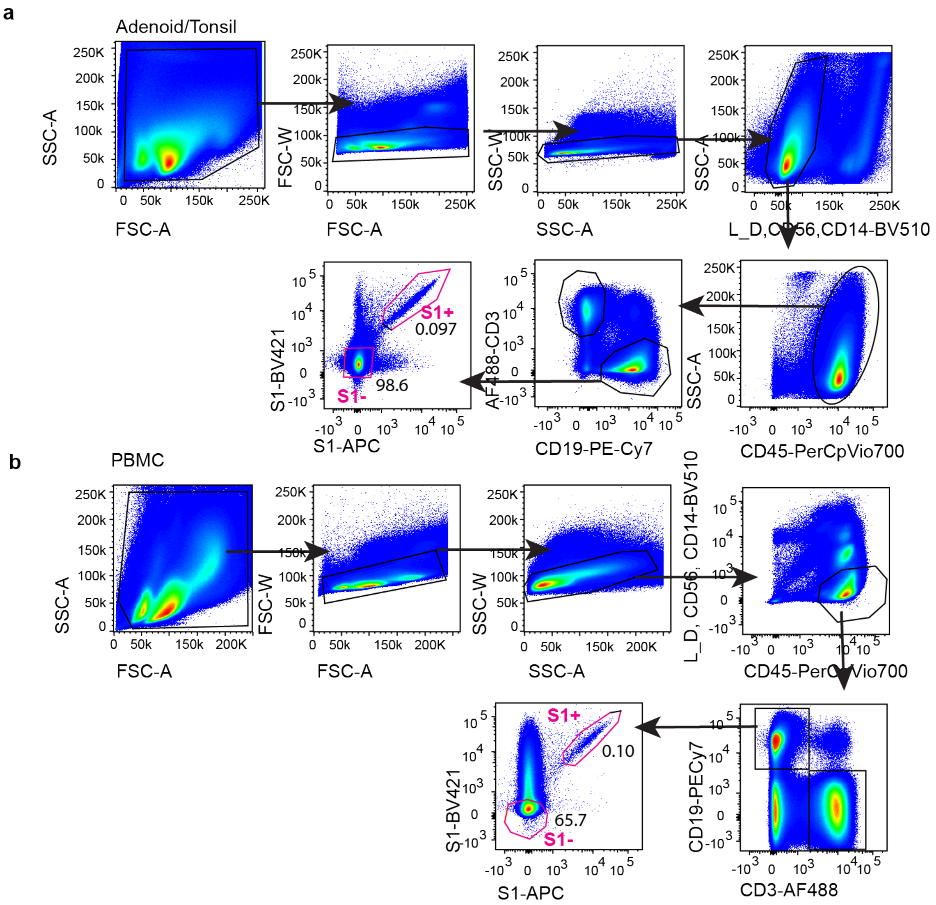

Supplementary Figure 3. Gating strategy for sorting S1⁺ and S1⁻ B cells used in CITE-seq and BCR sequencing analysis

Flow cytometry plots showing sorting strategy for S1 binding ($S1^+$) B cells (S1-BV421 and S1-APC double positive) and $S1^-$ B cells in (a) pharyngeal lymphoid tissues and (b) peripheral blood. Sorted cells were analyzed with CITE-seq and BCR sequencing.

Supplementary Figure 4

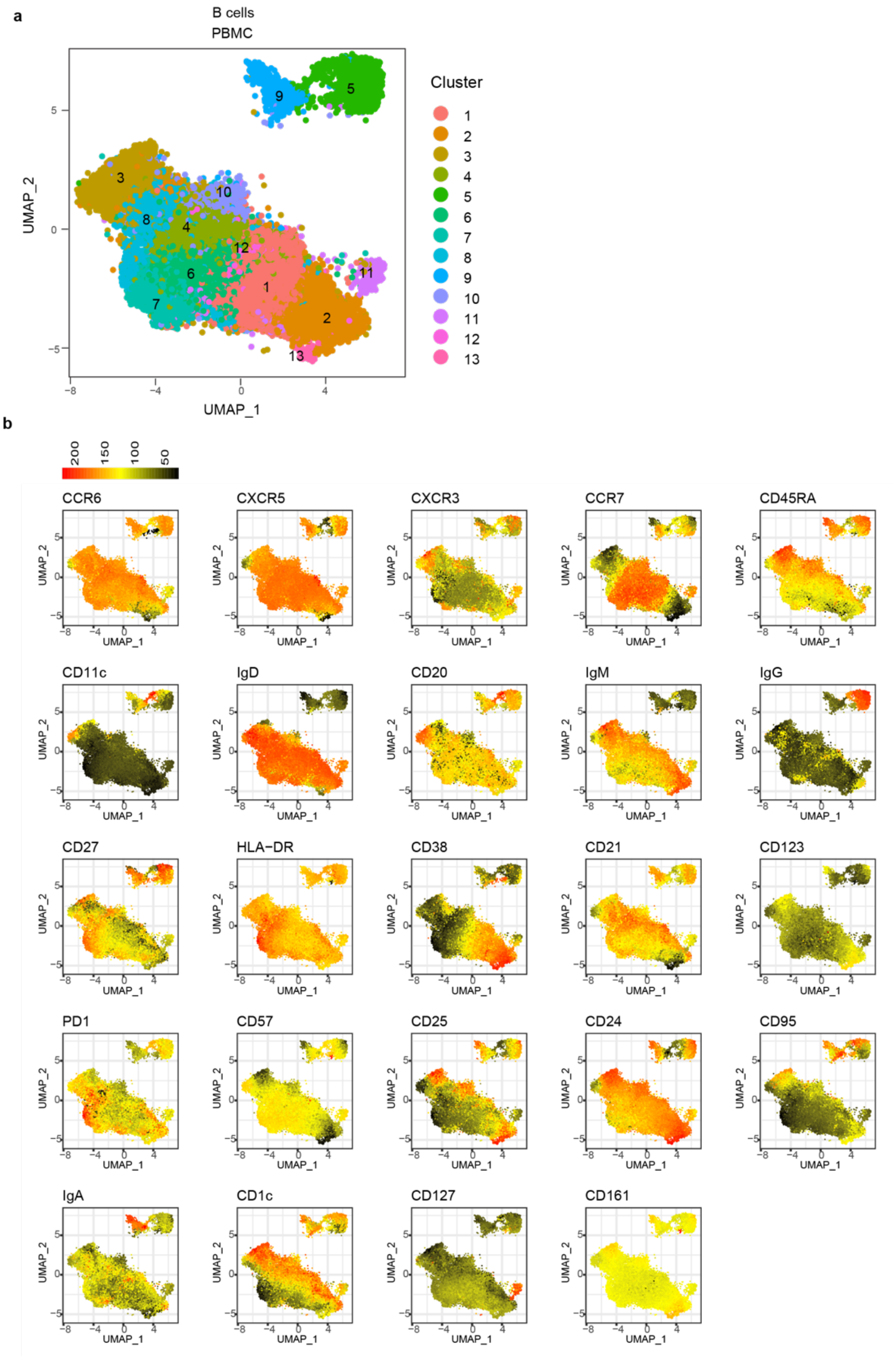

Supplementary Figure 4. UMAP of unsupervised clustering of B cells from PBMC

a. Uniform manifold approximation and projection (UMAP) of unsupervised clustering of surface markers from flow cytometric analysis of $CD19^+$ B cells from PBMC.

b. Heatmaps of marker/antibody expression overlayed on UMAP.

Supplementary Figure 5

T cell gating strategy for Adneoid and Tonsil

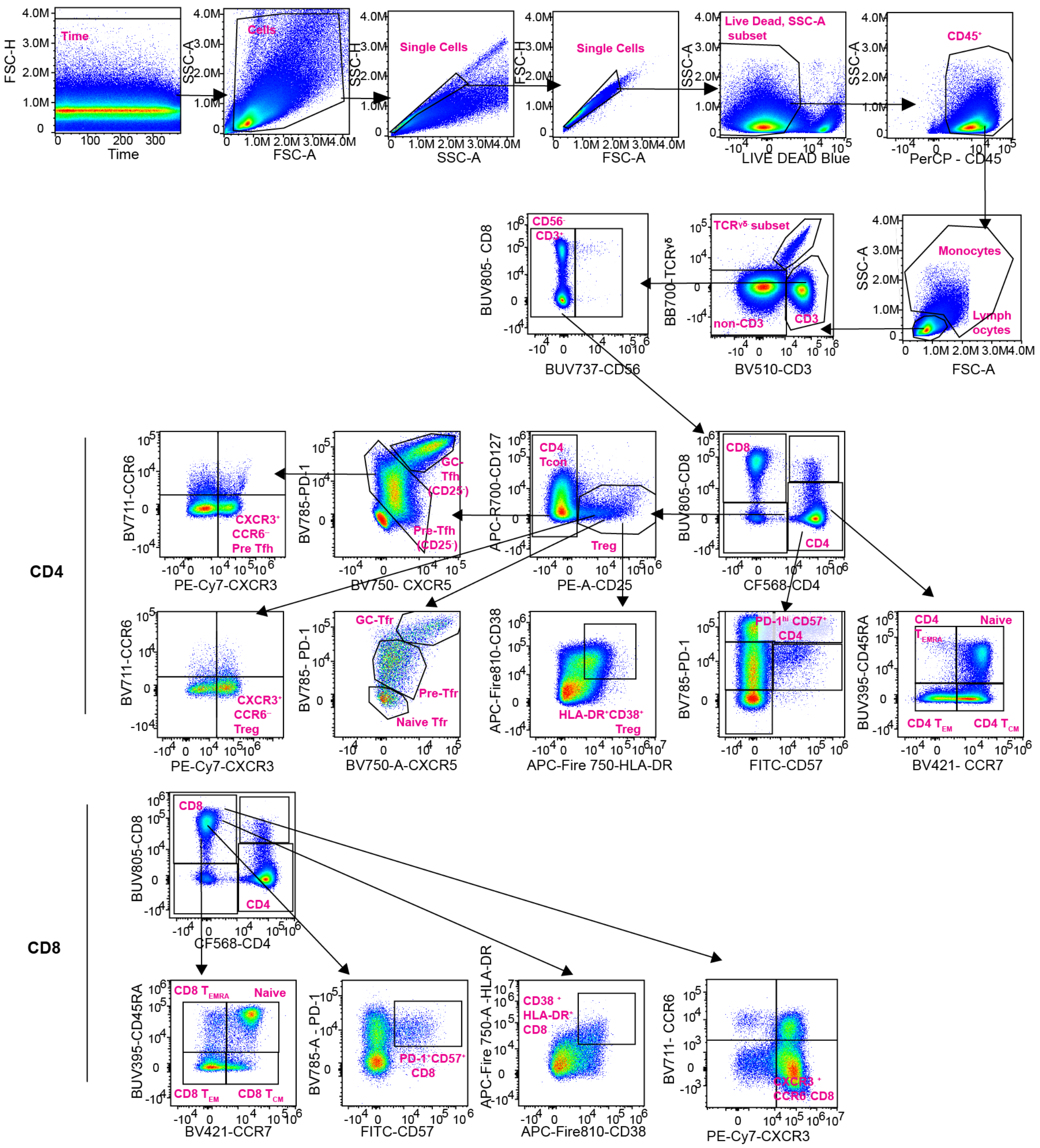

Supplementary Figure 5. Gating strategy of major CD4⁺ and CD8⁺ T cell populations in pharyngeal tissues.

Representative flow cytometry plots are shown. T_{EM} are effector memory T cells; T_{CM} are central memory T cells; T_{EMRA} are terminally differentiated effector memory T cells; Tfh are T follicular helper cells; Treg are regulatory T cells; and Tfr are T follicular regulatory cells.

Supplementary Figure 6

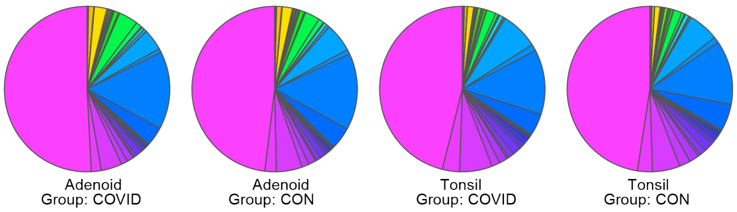

	IFN γ	IL2	IL10	IL17A	IL21	TNFα
1	+	+	+	+	+	+
2	+	+	+	+	-	+
3	+	+	+	-	+	+
4	+	+	+	-	-	+
5	+	+	-	+	+	+
6	+	+	-	+	+	-
7	+	+	-	+	-	+
8	+	+	-	+	-	-
9	+	+	-	-	+	+
10	+	+	-	-	+	-
11	+	+	-	-	-	+
12	+	+	-	-	-	-
13	+	-	+	+	+	+
14	+	-	+	+	+	-
15	+	-	+	+	-	+
16	+	-	+	+	-	-
17	+	-	+	-	+	+
18	+	-	+	-	+	-
19	+	-	+	-	-	+
20	+	-	+	-	-	-
21	+	-	-	+	+	+
22	+	-	-	+	+	-
23	+	-	-	+	-	+
24	+	-	-	+	-	-
25	+	-	-	-	+	+
26	+	-	-	-	+	-
27	+	-	-	-	-	+
28	+	-	-	-	-	-
29	-	+	+	+	+	+
30	-	+	+	+	+	-
31	-	+	+	+	-	+
32	-	+	+	+	-	-
33	-	+	+	-	+	+
34	-	+	+	-	+	-
35	-	+	+	-	-	+
36	-	+	+	-	-	-
37	-	+	-	+	+	+
38	-	+	-	+	+	-
39	-	+	-	+	-	+
40	-	+	-	+	-	-
41	-	+	-	-	+	+
42	-	+	-	-	+	-
43	-	+	-	-	-	+
44	-	+	-	-	-	-
45	-	-	+	+	+	+
46	-	-	+	+	+	-
47	-	-	+	+	-	+
48	-	-	+	+	-	-
49	-	-	+	-	+	+
50	-	-	+	-	+	-
51	-	-	+	-	-	+
52	-	-	+	-	-	-
53	-	-	-	+	+	+
54	-	-	-	+	+	-
55	-	-	-	+	-	+
56	-	-	-	+	-	-
57	-	-	-	-	+	+
58	-	-	-	-	+	-
59	-	-	-	-	-	+

Supplementary Figure 6. SPICE analysis of CD4$^+$ T cells from tonsil and adenoid

Pie charts show the proportion of responding CD4$^+$ T cells from adenoids and tonsils producing 59 different combinations of 6 cytokines (IFNγ, IL-2, IL-10, IL-17A, IL-21 and TNF-α) after PMA and ionomycin stimulation from COVID-19-convalescent donors (COVID) and controls (CON). (COVID adenoid n = 13, CON adenoid n = 13, COVID tonsil n = 13, CON tonsil n = 13). Frequencies were determined by Boolean combination gates in FlowJo and analyzed with SPICE. Combinations with frequencies below 0.01 from each donor were excluded from the analysis (categories IFNγ+IL2+IL10+IL17A+IL21+TNFα-, IFNγ+IL2+IL10+IL17A+IL21-TNFα-, IFNγ+IL2+IL10+IL17A-IL21+TNFα- and IFNγ+IL2+IL10+IL17A-IL21-TNFα- were excluded). Significance calculated using Mann-Whitney U test. Combinations with significant differences ($p<0.05$) in COVID vs. CON are highlighted in red.

Supplementary Figure 7

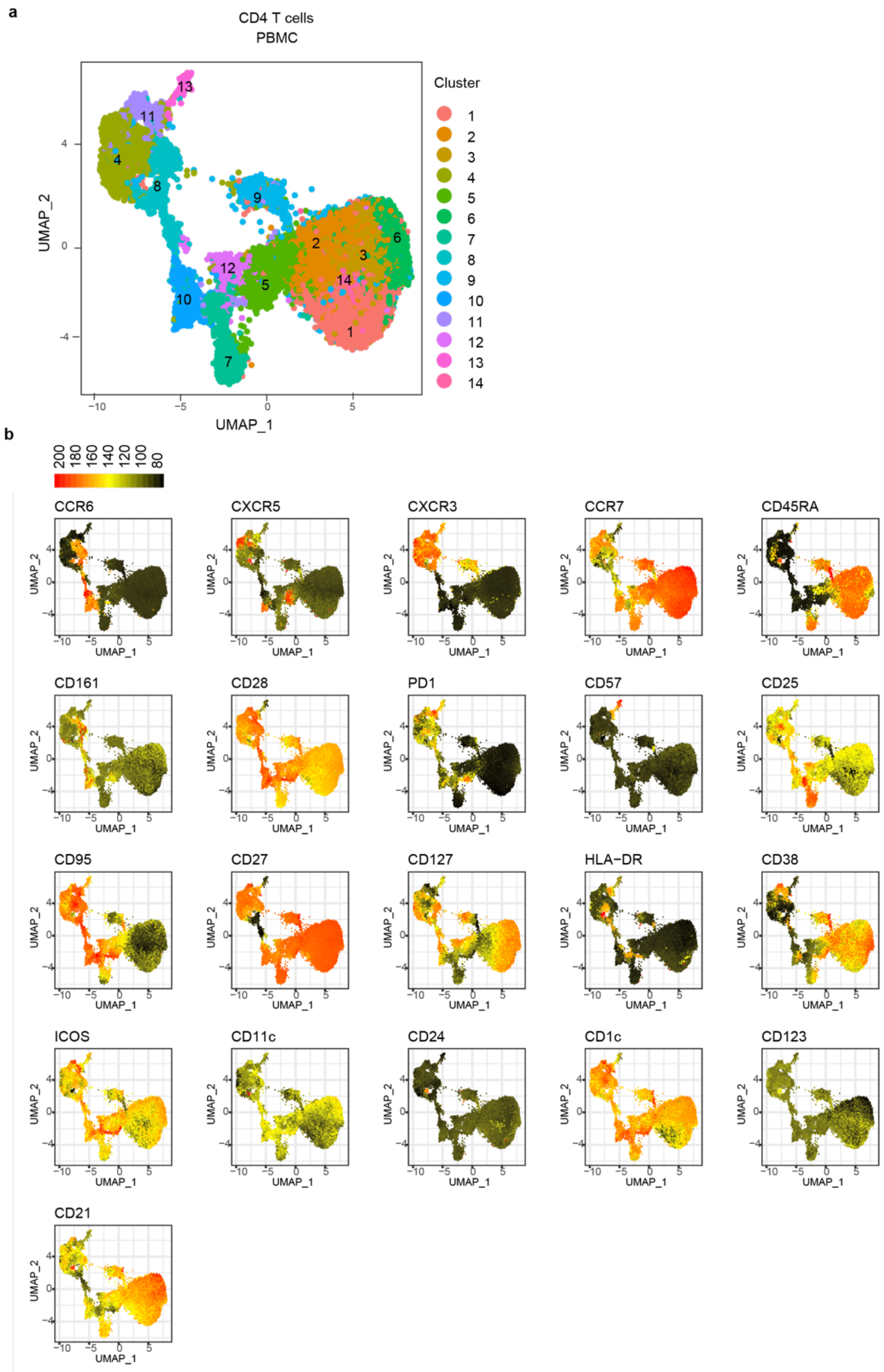

Supplementary Figure 7. UMAP of unsupervised clustering of CD4⁺ T cells from PBMC

a. Uniform manifold approximation and projection (UMAP) of unsupervised clustering of surface markers from flow cytometric analysis of CD4⁺ T cells from PBMC.

b. Heatmaps of marker/antibody expression overlayed on UMAP.

Supplementary Figure 8

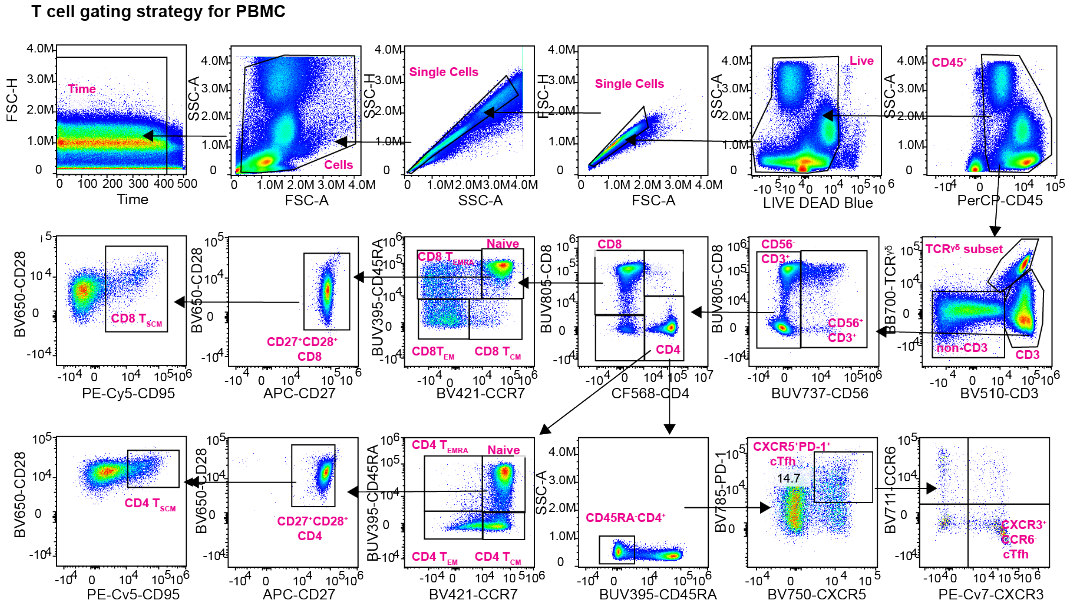

Supplementary Figure 8. Gating strategy of major CD4⁺ and CD8⁺ T cell populations in peripheral blood.

Representative flow cytometry plots are shown. T_{SCM} are T stem cell-like memory cells; T_{EM} are effector memory T cells; T_{CM} are central memory T cells; T_{EMRA} are terminally differentiated effector memory T cells; and cTfh are circulating T follicular helper cells.

Supplementary Figure 9

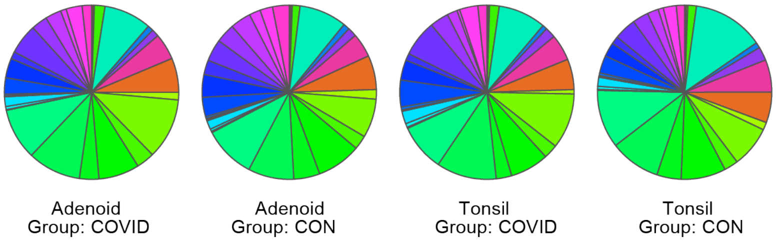

#	Gran	IFN γ	CD107a	IL2	TNFα
1	-	-	+	+	+
2	-	-	+	+	-
3	-	-	+	-	+
4	-	-	+	-	-
5	-	-	-	+	+
6	-	-	-	+	-
7	-	-	-	-	+
8	-	+	+	+	+
9	-	+	+	+	-
10	-	+	+	-	+
11	-	+	+	-	-
12	-	+	-	+	+
13	-	+	-	+	-
14	-	+	-	-	+
15	-	+	-	-	-
16	+	-	+	+	+
17	+	-	+	+	-
18	+	-	+	-	+
19	+	-	+	-	-
20	+	-	-	+	+
21	+	-	-	+	-
22	+	-	-	-	+
23	+	-	-	-	-
24	+	+	+	+	+
25	+	+	+	+	-
26	+	+	+	-	+
27	+	+	+	-	-
28	+	+	-	+	+
29	+	+	-	+	-
30	+	+	-	-	+
31	+	+	-	-	-

Supplementary Figure 9. SPICE analysis of CD8^{+} T cells from tonsil and adenoid

Pie charts show the proportion of responding CD8^{+} T cells from adenoids and tonsils producing 31 different combinations of 6 cytokines (granzyme B, IFN-γ, CD107a, IL-2 and TNF-α) after PMA and ionomycin stimulation from COVID vs. CON. Frequencies were determined by Boolean combination gates in FlowJo and analyzed with SPICE software. Combinations with frequencies below 0.01 from each donor were excluded from the analysis. Combinations with significant differences in COVID vs. CON are highlighted in red. Significance calculated using Mann-Whitney U test. Combinations with significant differences ($p<0.05$) in COVID vs. CON are highlighted in red.

Supplementary Figure 10

a

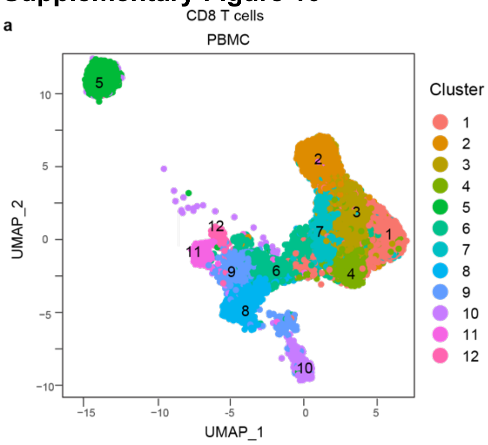

b

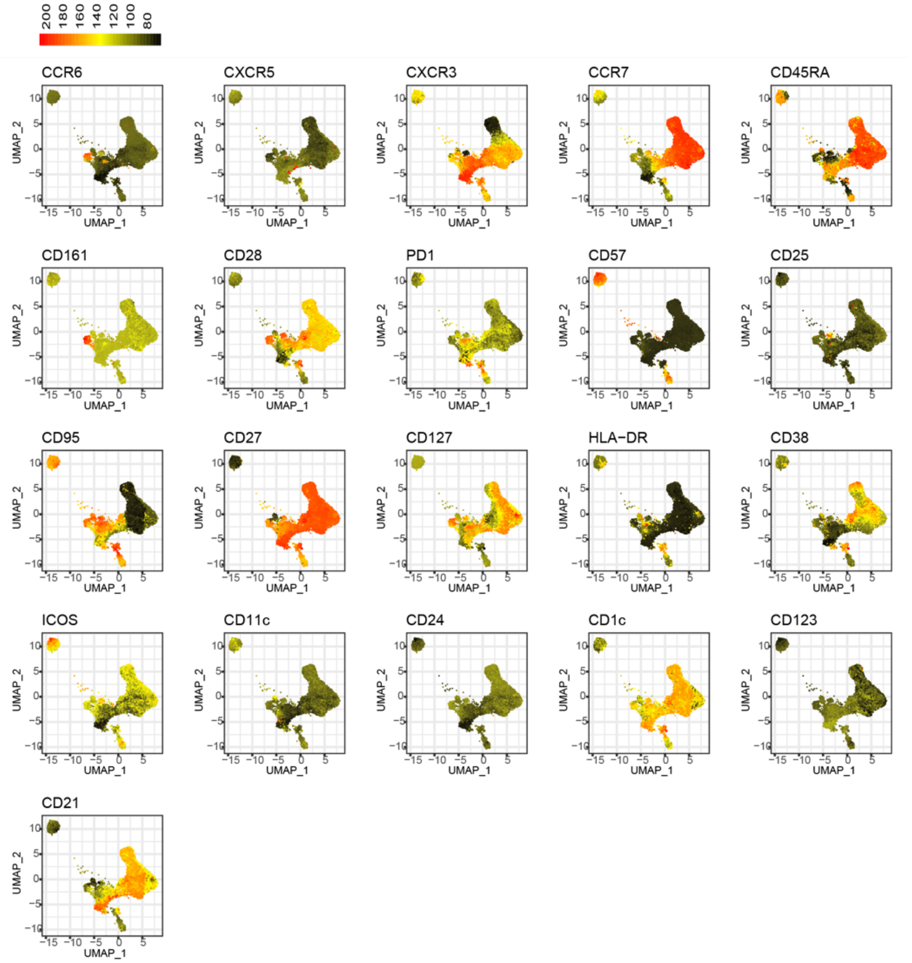

Supplementary Figure 10. UMAP of unsupervised clustering of CD8⁺ T cells from PBMC

a. Uniform manifold approximation and projection (UMAP) of unsupervised clustering of surface markers from flow cytometric analysis of $CD8^+$ T cells from PBMC.

b. Heatmaps of marker/antibody expression overlayed on UMAP.
